# eNOS Uncoupling, Shear‐Stress Tolerance, and the Two‐Threshold Model of Post‐Exertional Malaise in Long COVID: A Mechanistic Hypothesis With Implications for Physiotherapy and Recovery Protocols

**DOI:** 10.1111/micc.70082

**Published:** 2026-08-25

**Authors:** Yiannis K. Karipidis, Konstantinos Y. Karipidis

**Affiliations:** ^1^ Independent Researcher Giannitsa Greece; ^2^ Department of Physiotherapy, Faculty of Health Sciences International Hellenic University Alexander Campus Thessaloniki Greece

**Keywords:** endothelial dysfunction, eNOS uncoupling, long COVID, microvascular dysfunction, peroxynitrite, post‐exertional malaise, shear stress threshold, statin pleiotropic effects, tetrahydrobiopterin, two‐threshold model

## Abstract

**Objective:**

To propose and make testable a mechanistic hypothesis for post‐exertional malaise (PEM) in a clinically distinct subset of Long COVID patients, in whom delayed exertional symptoms coexist with consistently normal macrovascular investigations.

**Methods:**

Established vascular‐biology literature is synthesized into an integrated, falsifiable model centered on endothelial nitric oxide synthase (eNOS) uncoupling, from which mechanism‐specific predictions and a dynamic pre‐/post‐exertion biomarker validation framework are derived.

**Results:**

We propose that SARS‐CoV‐2‐induced endotheliitis activates inducible nitric oxide synthase and silently depletes the tetrahydrobiopterin (BH4) pool on return to activity, shear‐stress activation of structurally intact eNOS against a depleted BH4 background yields superoxide rather than nitric oxide, generating peroxynitrite that sustains a self‐amplifying nitro‐oxidative cycle. The Two‐Threshold Model distinguishes a PEM threshold from a shear‐stress‐tolerance threshold and predicts that prolonged immobility may paradoxically erode endothelial function. eNOS uncoupling is positioned as one node among alternative microvascular pathways, and autonomic findings are proposed to be secondary within this phenotype.

**Conclusions:**

This phenotype‐specific, falsifiable hypothesis yields mechanism‐derived predictions and rehabilitation implications consistent with symptom‐contingent pacing guidance; it does not claim to explain all Long COVID presentations.

## 1 Introduction

Long COVID affects an estimated 65 million individuals worldwide [1]. A clinically distinct subset presents with a specific pattern: post‐exertional malaise with 12–48 h delayed onset, chest tightness and dyspnea appearing at rest the morning after activity, suppressed HRV, cold extremities, and brain fog with consistently normal results on standard cardiovascular investigations: echocardiogram, exercise stress test, and coronary CT angiography.

This diagnostic pattern is not incidental. Conventional diagnostic tools are designed to detect pathology in epicardial coronary arteries and are not sensitive to dysfunction at the level of the coronary microcirculation (arterioles and capillaries < 300 μm). Patients within the proposed vascular‐PEM phenotype may repeatedly demonstrate normal macrovascular findings despite ongoing symptoms consistent with impaired tissue perfusion. This may reflect the limitations of diagnostic resolution rather than absence of pathology. Modalities with potential relevance include PET coronary flow reserve, cardiac stress MRI, flow‐mediated dilation (FMD), post‐occlusive reactive hyperaemia (PORH) with finger thermal monitoring, and near‐infrared spectroscopy (NIRS)‐derived tissue oxygenation indices, none of which was designed for this specific context and whose sensitivity for the proposed phenotype remains to be established. Importantly, the proposed mechanism is not anatomically restricted to the coronary circulation. Dysfunctional endothelium and the resulting eNOS uncoupling cycle may occur in any microvascular bed—cerebral, renal, skeletal muscle, or peripheral—producing symptom constellations that differ by location but share the same upstream biochemical mechanism. The clinical expression may vary depending on which microvascular territories are most affected in a given individual, while the proposed biochemical driver BH4/dihydrobiopterin (BH2) imbalance and eNOS uncoupling is hypothesized to remain a shared upstream mechanism. These organ‐specific associations are proposed as mechanistically plausible but require independent prospective investigation.

A downstream consequence of this diagnostic gap may be misattribution to dysautonomia, anxiety, or deconditioning. Within this model, apparent autonomic dysfunction is proposed as a secondary consequence of microvascular endothelial failure: NO deficiency impairs peripheral vasodilation, driving compensatory sympathetic activation. The proposed causal link between eNOS‐derived NO and autonomic balance has biochemical precedent: NO has been shown to act as a sympatho‐inhibitory and vagotonic neuromodulator at both central and peripheral levels, with eNOS‐derived NO reinforcing presynaptic vagal facilitation in cardiac myocytes [2]. Within this framework, the suppressed HRV consistently observed in the vascular‐PEM phenotype may not represent primary autonomic disease but rather a downstream functional consequence of the same eNOS uncoupling driving microvascular dysfunction. Managing the autonomic manifestation without addressing the proposed endothelial cause may therefore produce temporary symptomatic relief followed by rebound.

The mechanistic trigger for exercise‐induced PEM remains undefined in current literature. Charlton et al. [3] state directly that “the mechanistic trigger for physical exercise to induce PEM is unknown”, while identifying endothelial abnormalities as a contributing factor alongside mitochondrial dysfunction. Haunhorst et al. [4] describe reduced systemic oxygen extraction mediated by microvascular dysfunction without specifying the biochemical mechanism. We propose functional eNOS uncoupling driven by BH4/BH2 imbalance as a candidate mechanism that may explain the most distinctive features of PEM: delayed onset, disproportionality to the triggering activity, and self‐amplifying severity. To appreciate the clinical weight of this diagnostic and mechanistic gap, the lived experience of the vascular‐PEM phenotype warrants brief description at the outset. This is not a syndrome that manifests only during physical activity. The affected individual wakes each morning already operating at a fraction of their prior functional capacity in most activities of daily living. Breathing feels effortful despite the absence of any structural abnormality. Concentration becomes difficult. Simple everyday movements require effort. For many, the most physically demanding activity of the day is a short walk to the bathroom. For the athlete who once trained daily, the collapse is total: the body that carried them through long efforts now cannot sustain a 10‐min walk without consequence. For the person who never trained at all, the consequences are no less severe: ordinary domestic activity, a conversation sustained too long, or mild emotional stress may each be sufficient to provoke the same delayed collapse. The most feared feature is post‐exertional malaise: a collapse 12–48 h after activity, characterized by systemic flu‐like malaise, cognitive disintegration, and profound fatigue disproportionate to any recognizable exertion. This is not deconditioning. The mechanism has been unknown. A further burden carried by this population is systematic misattribution. Patients who present with chest tightness, dyspnea, palpitations, and cognitive impairment whose cardiac investigations return entirely normal are frequently told that their symptoms are anxiety, deconditioning, or medically unexplained. This misattribution is not the result of clinical negligence: it is the result of investigating macrovascular structure with tools designed for it, while the actual disease process unfolds in blood vessels too small for those tools to resolve. The standard investigations are negative not because nothing is wrong, but because the wrong scale is being examined. This distinction between the absence of pathology and the absence of detectable pathology is foundational to the framework presented here. The search for a mechanistic explanation did not originate in a laboratory. It emerged from systematic investigation by the lead author, a former competitive endurance athlete who developed this phenotype following SARS‐CoV‐2 infection. The search for an explanation was grounded in the analysis of longitudinal biometric data collected before and after illness, identification of consistent patterns of failure and partial recovery, and engagement with the physiological and biochemical literature. The convergence of these lines of evidence toward the eNOS uncoupling hypothesis presented here is offered not as a proven mechanism but as a falsifiable hypothesis grounded in established vascular biochemistry, with the aim of providing a testable framework from which a precise, individually calibrated management protocol for this phenotype may eventually be derived.

## 2 From Endothelial Dysfunction to Diagnostic Failure: The Proposed Mechanistic Chain

The proposed mechanism operates as a sequential chain in which each step may create the conditions for the next, potentially connecting the initial viral insult to the observable clinical syndrome.

### 2.1 Phase 1: Endothelial Priming

SARS‐CoV‐2 binds ACE2 receptors on endothelial cells, inducing endotheliitis [1]. While SARS‐CoV‐2 is the index trigger in this model, the proposed cascade may be relevant to endotheliitis arising from other viral or systemic inflammatory insults in individuals with underlying endothelial or redox vulnerability including those with preexisting genetic variants affecting antioxidant defense or BH4 metabolism. NF‐κB activation may increase reactive oxygen species (ROS) production, oxidizing BH4 to BH2. ACE2 downregulation may shift vascular tone toward Angiotensin II dominance, which has been shown to promote eNOS uncoupling through RhoA/Rho‐kinase/arginase pathways [5]. Inflammatory iron sequestration by the reticuloendothelial system may elevate labile iron, catalyzing the Fenton reaction (Fe^2+^ + H_2_O_2_ → Fe^3+^ + ˙OH + OH^−^) and providing an additional upstream source of BH4‐oxidizing radicals [6].

#### 2.1.1 The Proposed iNOS–eNOS Transition

A mechanistically important distinction is proposed between the roles of iNOS and eNOS across the phases of this syndrome. During and immediately following acute SARS‐CoV‐2 infection, NF‐κB‐driven iNOS induction in macrophages and vascular smooth muscle may generate sustained high‐output NO alongside concurrent ROS conditions favoring peroxynitrite formation and progressive BH4 oxidation. This iNOS‐mediated BH4 depletion may proceed silently during apparent clinical recovery: as the patient rests, no exertional shear stress is applied, and the depleted BH4 state generates no overt symptoms. Upon return to activity weeks to months later, shear stress activation of eNOS structurally intact but operating against a chronically depleted BH4 background may convert the latent biochemical deficit into the first overt PEM crash. The iNOS phase thus primes the endothelium; the eNOS uncoupling phase generates the characteristic delayed exertional syndrome. A critical clinical implication of this temporal model is that the progressive decline in functional capacity observed during the apparent recovery period commonly attributed to deconditioning or post‐viral fatigue may itself reflect the ongoing silent biochemical process. Each exertional bout in the BH4‐depleted state, however modest, may generate peroxynitrite rather than NO, progressively lowering the threshold for subsequent dysfunction. The patient who perceives themselves as recovering and attempts cautious resumption of activity may in fact be accelerating the depletion cycle. This applies equally to the trained athlete and the previously sedentary individual: the mechanism is identical, though the timeline to overt PEM crash may differ due to the athlete's larger pre‐illness BH4 reserve a buffer that delays but does not prevent the eventual threshold crossing. This distinction also clarifies the contraindication for NO‐substrate supplementation: in the presence of active iNOS, L‐arginine may increase peroxynitrite generation; with uncoupled eNOS, the same substrate may increase superoxide production the Arginine Paradox operating through two independent pathways simultaneously. A mechanistically important clinical corollary applies specifically to endurance athletes who develop this phenotype following infection. Prior to illness, sustained training generates high laminar shear, upregulating eNOS expression and expanding functional eNOS protein density. Following iNOS‐mediated BH4 depletion, this larger pool of uncoupled eNOS may generate proportionally greater superoxide per exercise bout than in nonathletes. Each training session that appeared to produce normal adaptation before illness may instead produce peroxynitrite in a BH4‐depleted endothelium, progressively depleting the BH4 pool further and lowering the PEM threshold with each session. VO_2_max may fall progressively not from deconditioning but from worsening eNOS‐endothelial mismatch, until the BH4 pool reaches a critical threshold and the first overt PEM crash occurs often interpreted as overtraining or sudden deconditioning rather than recognized as the endpoint of a months‐long subclinical biochemical process. Persistent viral antigen reservoirs or ONOO^−^‐mediated IkB kinase nitration may sustain low‐grade NF‐κB and iNOS activity beyond the acute phase, maintaining background BH4 depletion even in patients without demonstrable ongoing infection. Within the proposed model, this low‐grade iNOS activity is not limited to the acute illness phase but is proposed to continue at reduced intensity throughout the Long COVID period, providing a mechanistic basis for the resting pseudo‐hypoxic symptoms breathlessness at rest, air hunger, cognitive impairment that characterize this phenotype independently of exertion. The dual role of iNOS and eNOS in acute SARS‐CoV‐2 infection has been reviewed by Guimarães et al. [7], who document concurrent eNOS uncoupling and iNOS overactivation in pulmonary vascular beds during acute disease. The present model extends this framework to the Long COVID context by proposing that the temporal separation between the iNOS priming phase and the subsequent exertion‐triggered eNOS uncoupling phase rather than their simultaneous activation may be the mechanistic basis for the characteristic delayed‐onset exertional syndrome not observed during or immediately following the acute infection.

#### 2.1.2 Pseudo‐Hypoxia and the Warburg Effect

A further mechanistic consequence of sustained iNOS‐derived NO excess is proposed competitive inhibition of mitochondrial Complex IV (cytochrome c oxidase), which shares its binding site between O_2_ and NO. At the elevated NO concentrations generated by iNOS overactivation, this competitive displacement may produce cellular pseudo‐hypoxia, a state in which oxygen delivery is adequate but mitochondrial utilization is impaired, producing a normal pulse oximetry reading despite intracellular energy deficit. Pseudo‐hypoxia activates HIF‐1α, which redirects pyruvate away from mitochondrial oxidative phosphorylation toward cytosolic aerobic glycolysis (the Warburg effect), generating ATP at approximately 5% of normal mitochondrial efficiency while producing lactate. This may contribute to the severe resting fatigue and disproportionate post‐exertional symptom amplification characteristic of this phenotype, independently of exertional load. A mechanistically important secondary consequence of HIF‐1α activation is proposed suppression of GCH1 expression, the rate‐limiting enzyme for BH4 de novo synthesis, potentially accelerating BH4 pool depletion during the iNOS phase and compressing the interval before eNOS uncoupling becomes clinically manifest following return to activity.

### 2.2 Phase 2: BH2 Competitive Displacement

BH4 and BH2 share the same cofactor binding site on eNOS. As the BH4/BH2 ratio falls, BH2 may increasingly occupy this site. The enzyme remains structurally intact and is activated normally by shear stress, but with BH2 at the cofactor site, the electron transfer cycle may not complete NO synthesis, potentially diverting electrons to molecular oxygen and generating superoxide. This proposed mechanism represents not eNOS absence but eNOS misdirection [8, 9, 10].

### 2.3 Phase 3: DHFR Inhibition—The Proposed Reason for Self‐Perpetuation

Dihydrofolate reductase (DHFR) is responsible for recycling BH2 back to BH4, using 5‐methyltetrahydrofolate (5‐MTHF) as a cofactor. Peroxynitrite has been shown to nitrate proteins at critical tyrosine residues in oxidative environments [6]. If this process affects DHFR, each mobilization event that tips into uncoupling may leave the BH4/BH2 ratio worse than before, progressively lowering the threshold for subsequent events. This proposed positive‐feedback loop may provide a mechanistic framework for the characteristic worsening trajectory observed in this phenotype, though direct demonstration in Long COVID patients remains to be established. A mechanistically important temporal question merits direct address: the plasma half‐life of BH4 is approximately 4 h, which appears inconsistent with the characteristic 12–48 h delay before PEM onset. However, this dissociation is explicable without invoking sustained BH4 depletion as the proximate cause of delayed symptoms. Within the proposed model, BH4 depletion functions as the initiating trigger for a downstream cascade whose kinetics substantially outlast the cofactor deficit itself. First, peroxynitrite generated during the uncoupling event activates NF‐κB through IκB kinase nitration, a transcriptional pathway with an inherent delay of 6–18 h before measurable iNOS upregulation and renewed oxidative BH4 consumption. Second, peroxynitrite‐mediated nitration of DHFR is a posttranslational modification that accumulates progressively and is not reversed within hours; each uncoupling event therefore deposits cumulative DHFR damage that impairs subsequent BH4 recycling capacity independently of the acute BH4 level. Third, mitochondrial Complex I and III damage by peroxynitrite generates sustained secondary ROS production that continues to amplify the nitro‐oxidative environment after the initial exercise bout has ended. This is further compounded by the post‐exertional cessation of laminar flow: entering a state of immobility removes the mechanical stimulus for KLF2 expression and GCH1‐driven BH4 synthesis, thereby withdrawing the principal endogenous defense against progressive BH4 pool depletion during the recovery window [11]. The net result is that PEM represents the clinical manifestation of the downstream inflammatory and mitochondrial consequences of the initiating uncoupling event, consequences whose peak expression at 12–48 h reflects the kinetics of transcriptional activation, protein nitration accumulation, and mitochondrial dysfunction rather than the kinetics of BH4 itself. This temporal model also predicts that BH4 or BH4/BH2 ratio measurement immediately post‐exertion may underestimate the degree of ongoing uncoupling, and that the 24–48 h sampling window proposed in the biomarker validation framework (Section 7) is mechanistically justified rather than arbitrary.

**FIGURE 1:**
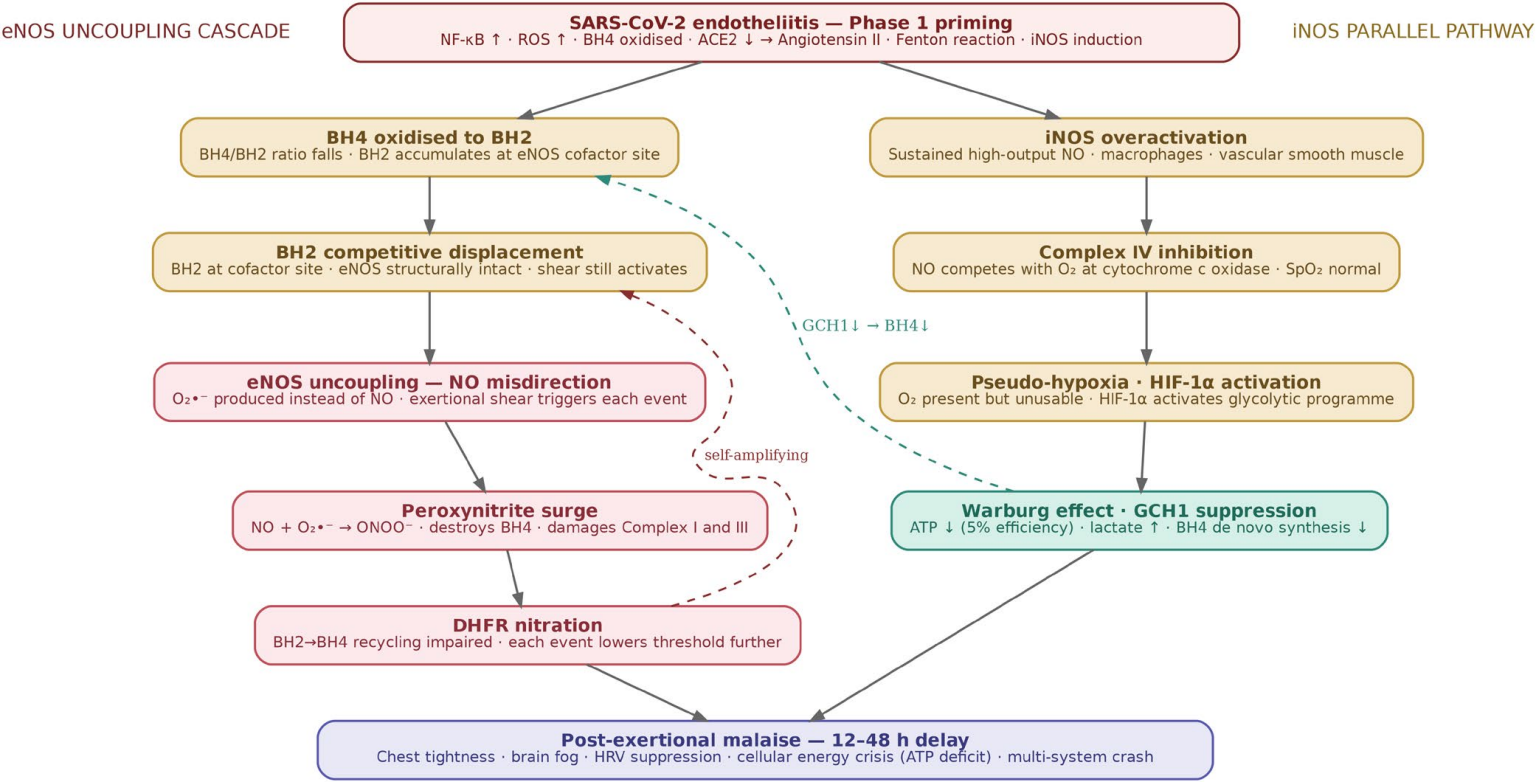
Proposed dual‐pathway mechanistic cascade from SARS‐CoV‐2 endotheliitis to post‐exertional malaise. Two parallel pathways arise from a shared Phase 1 priming event (top box). Left column eNOS uncoupling cascade: Progressive BH4 oxidation to BH2; competitive BH2 displacement at the eNOS cofactor site; functional eNOS uncoupling with superoxide (O_2_^−^) production upon shear stress activation; peroxynitrite surge (NO + O_2_^−^ → ONOO^−^) with simultaneous BH4 destruction and mitochondrial Complex I/III damage; and dihydrofolate reductase (DHFR) nitration impairing BH2‐to‐BH4 recycling. The dashed loop on the left margin illustrates the proposed self‐amplifying mechanism: each PEM‐threshold crossing deposits cumulative DHFR damage, progressively lowering the BH4/BH2 ratio and reducing the threshold for subsequent events. Right column iNOS parallel pathway: NF‐κB‐driven iNOS induction generating high‐output NO in macrophages and vascular smooth muscle; competitive inhibition of mitochondrial Complex IV by NO, producing cellular pseudo‐hypoxia despite normal pulse oximetry; HIF‐1α activation driving aerobic glycolysis (Warburg effect) at approximately 5% of normal ATP efficiency with concurrent lactate accumulation; and HIF‐1α‐mediated suppression of GCH1 expression, reducing de novo BH4 synthesis capacity. The teal cross‐connection arrow illustrates the proposed interaction between pathways: GCH1 suppression from the iNOS branch accelerates BH4 pool depletion in the eNOS cascade, compressing the interval before uncoupling becomes clinically manifest upon return to activity. Both pathways converge on the lower box: post‐exertional malaise appearing 12–48 h after the triggering activity, comprising chest tightness, brain fog, HRV suppression, and cellular energy crisis (ATP deficit).

Figure 1 summarizes the proposed dual‐pathway mechanistic cascade described above.

### 2.4 Phase 4: The Proposed Diagnostic Invisibility

The proposed clinical picture presents a diagnostic paradox. Microvascular endothelial dysfunction may produce symptoms of chest tightness, dyspnea, palpitations, cold extremities, headache, brain fog, gastrointestinal discomfort, or limb weakness that direct clinicians toward investigations of the organ system most prominently implicated: cardiac investigations for chest tightness, pulmonary investigations for dyspnea, neurological investigations for cognitive impairment, vascular investigations for cold extremities. Each investigation returns normal because it interrogates the macrovascular territory of the relevant organ rather than the shared microvascular endothelium that the present model proposes as the common upstream source.

The apparent autonomic abnormalities are proposed to be real but secondary: representing cardiovascular compensation for inadequate peripheral vasodilation in a NO‐deficient microvascular bed. When the endothelium cannot produce NO on demand, heart rate and contractility may increase to maintain perfusion against elevated peripheral resistance. This compensatory response may then be interpreted as primary dysautonomia. The proposed causal frame is: endothelial dysfunction → compensatory sympathetic activation → apparent dysautonomia. The intermediate mechanisms including impaired peripheral vasodilation, altered microvascular distribution of cardiac output, and reduced NO‐mediated vascular tone regulation are multiple and not reducible to a single step, but share the same upstream endothelial failure. If this framing is correct, managing the autonomic manifestation alone would produce temporary improvement followed by recurrence. This proposal is specific to the vascular‐PEM phenotype and does not dispute that primary autonomic disorders including postural orthostatic tachycardia syndrome are well documented in Long COVID and may predominate in other phenotypes. A further manifestation of this proposed diagnostic invisibility is episodic dyspnea occurring at rest without exertion. Within the present model, the iNOS‐mediated pseudo‐hypoxia and Warburg‐mediated metabolic shift described in Section 2.1 drive continuous aerobic glycolysis at rest, generating lactate and producing mild fluctuating metabolic acidemia. This is sufficient to stimulate central and peripheral chemoreceptors, driving a subjective sensation of air hunger in the presence of normal pulse oximetry, spirometry, and arterial oxygen tension. This pattern may explain the clinically frustrating mismatch between severe episodic breathlessness and consistently normal respiratory investigations in this phenotype—a mismatch that, like the cardiac diagnostic gap, may reflect the limitations of investigations designed to detect structural or gas‐exchange pathology rather than intracellular metabolic dysfunction. A proposed contributing factor to the episodic rather than continuous nature of this symptom is the fluctuating background iNOS activity driven by dietary and postural shear changes, psychological stress, and thermal variation throughout the day, each transiently amplifying lactate accumulation and chemoreceptor stimulation before subsiding.

## 3 The Two‐Threshold Model

A proposed central contribution of this model is the distinction between two mechanistically independent thresholds. These are presented as operational constructs derived from the proposed mechanism, not as validated physiological entities and require prospective study.

*The PEM threshold* is proposed as the level of exertional load at which the nitro‐oxidative cascade may transition from a subclinical perturbation into a self‐amplifying loop. If this threshold is crossed, peroxynitrite generation may exceed endothelial repair capacity, producing DHFR nitration, sustained BH4 depletion, and the characteristic delayed multisystem crash. Within this model, every crossing may lower the threshold further by leaving the BH4 pool more depleted than before.

*The shear stress tolerance threshold* is proposed as the minimum sustained laminar shear stimulus required to maintain GTP cyclohydrolase I (GTPCH‐1) Ser81 phosphorylation and ongoing BH4 de novo synthesis in the vascular endothelium. Widder et al. [12] demonstrated that laminar, but not oscillatory, shear stress induces GTPCH‐1 Ser81 phosphorylation in human aortic endothelial cells, increasing BH4 production by up to 30‐fold. Based on this established mechanism, the present model proposes that this physiological signal requires consistent maintenance to prevent progressive BH4 pool depletion in the absence of physical activity.

The proposed paradox of complete rest in this phenotype is that removing all ambulatory activity may also remove the principal physiological maintenance signal for endothelial BH4 synthesis. The KLF2 → GCH1 → BH4 axis depends on sustained laminar shear for activation. In complete inactivity, this signaling may decline, KLF2 expression may fall, GCH1 activity may decrease, and existing BH4 may be progressively oxidized without replacement.

**FIGURE 2:**
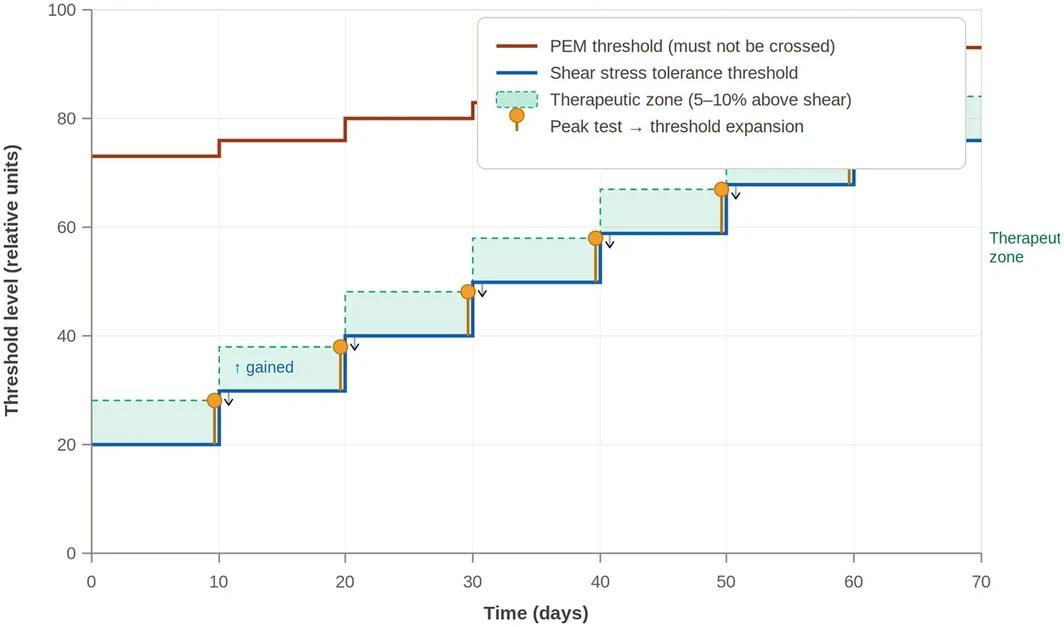
The Two‐Threshold Model: proposed progressive expansion of the shear stress tolerance threshold and PEM threshold through structured, hemodynamically‐informed, symptom‐contingent activity management. The PEM threshold (coral stepped line) represents the exertional ceiling above which the nitro‐oxidative cascade becomes self‐amplifying; this threshold must never be crossed. The shear stress tolerance threshold (blue stepped line) represents the minimum laminar shear stimulus required to sustain GTPCH‐1‐driven BH4 synthesis; this threshold must be consistently maintained. The therapeutic zone (teal shaded area) is a narrow activity band 5%–10% above the shear stress threshold within which laminar shear‐mediated BH4 synthesis is proposed to occur without crossing the PEM threshold. Amber markers indicate periodic biomarker‐gated threshold‐testing sessions at approximately 10‐day intervals, attempted only during stable periods with no PEM events and stable RMSSD recovery kinetics, during which session duration or pace is cautiously probed to the top of the therapeutic zone without systematic escalation. This is mechanistically distinct from graded exercise therapy (GET): escalation is not scheduled but contingent on individual biomarker response; the total activity envelope is never increased beyond the individual's pacing‐defined symptom ceiling. Each threshold test successfully completed without delayed multisystem worsening (confirmed PEM) produces an upward step in both thresholds representing the proposed mechanism by which a new, higher shear stress tolerance and PEM ceiling are established. Progressive ascent of both thresholds over time reflects the cumulative signal of sustained laminar shear exposure and improving eNOS coupling capacity under concurrent Layer 1–4 pharmacological support. All rehabilitation guidance is presented as hypothesis‐derived and requires prospective clinical validation before implementation.

The Two‐Threshold Model is illustrated in Figure 2.

### 3.1 The Heart Rate Paradox: Hemodynamic Quality as an Independent Variable

Heart rate alone may be an insufficient safety marker in the vascular‐PEM phenotype. Two activity patterns may produce identical heart rates and remain below the same metabolic threshold (LT1), yet generate mechanistically different hemodynamic environments at the microvascular endothelium.

Sustained constant‐pace walking on flat, level ground generates steady, unidirectional laminar shear in the physiologically protective range (10–20 dyn/cm^2^) [13]. Widder et al. [12] demonstrated that this pattern activates GTPCH‐1 via Ser81 phosphorylation, supports BH4 synthesis, and may maintain eNOS in a coupled configuration. Interval or variable‐pace activity at the same average heart rate generates oscillatory and turbulent shear (0–5 dyn/cm^2^ with bidirectional components), activating nicotinamide adenine dinucleotide phosphate (NADPH) oxidase, potentially suppressing GTPCH‐1 phosphorylation, and possibly accelerating BH4 depletion [12].

This distinction may help explain a clinically confusing observation: why some patients tolerate a 30‐min slow walk without delayed symptoms but develop post‐exertional worsening after brief intervals at the same average heart rate and even at the same peak heart rate. Within this model, the intervals may generate transient velocity spikes creating oscillatory shear in microvascular bifurcations, tipping an already‐depleted system toward uncoupling, while the slow walk may generate sustained laminar shear supporting BH4 resynthesis. Both sessions appear identical on a heart rate monitor but may be biochemically distinct.

## 4 Functional Phenotypes Predicted by the Two‐Threshold Model and Their Rehabilitation Implications

If the proposed model is correct, clinical heterogeneity in Long COVID‐associated PEM may reflect differing relative positions of two dynamic physiological boundaries: (1) the exertional threshold above which nitro‐oxidative amplification may be triggered (PEM threshold), and (2) the minimum laminar shear exposure proposed to maintain endothelial BH4 homeostasis (shear tolerance threshold). Variation in these thresholds may generate apparently contradictory exercise responses across patients while remaining mechanistically coherent within the same framework.

These phenotype descriptions are presented as follows:

*Hypothesis‐generating clinical patterns*, not validated diagnostic classifications. A foundational point applies across all phenotypes: the impairment described in this phenotype is not confined to moments of exertion. All four phenotypes share a common baseline of reduced daily function, diminished cognitive capacity, episodic breathlessness at rest, persistent fatigue, and attenuated physiological reserve that reflects the continuous iNOS‐driven pseudo‐hypoxia and Warburg‐mediated energy deficit described in Section 2.1. The phenotypic distinctions below describe how each subgroup responds to exertional load imposed upon this already‐compromised baseline, not the absence of symptoms at rest.

### 4.1 Phenotype A: Severely Reduced Continuous Tolerance

Some patients may tolerate only brief periods of upright activity before developing chest tightness, dyspnea, or delayed worsening. Within the present framework, this pattern may reflect critically reduced shear tolerance combined with a low PEM threshold. In such cases, uninterrupted activity may exceed endothelial compensatory capacity before beneficial laminar signaling has been sustained long enough to support BH4 synthesis.

This model predicts that fractionated low‐dose movement, separated by recovery intervals, may be better tolerated than continuous activity in this subgroup. This prediction requires formal testing and should not be implemented as a clinical protocol without specialist evaluation.

### 4.2 Phenotype B: Moderate Continuous Tolerance With Delayed Deterioration

Other patients may tolerate 30–45 min of low‐intensity continuous movement but deteriorate later the same day or the following morning. In this framework, baseline endothelial reserve may be partially preserved, but delayed post‐exertional oxidative processes may progressively impair BH4 availability after cessation of activity. Within this model, delayed worsening in Phenotype B arises from two concurrent mechanisms. First, the abrupt transition from active flow to inactivity may generate a brief oscillatory flow transient that, in a BH4‐depleted endothelium, could initiate the uncoupling cascade. Second, and independently, the exercise session itself may have generated active NO misdirection: with BH4 partially depleted, the shear‐activated eNOS may have produced superoxide and peroxynitrite rather than NO during the session, initiating a delayed nitro‐oxidative cascade that manifests as PEM 12–48 h later even if the patient felt well immediately afterwards. Both mechanisms may operate simultaneously and reinforce each other in this subgroup.

This model predicts that gradual cool‐down with maintained low‐velocity movement, avoidance of abrupt postexercise inactivity, and distributed daily activity may be better tolerated than clustered activity loads.

### 4.3 Phenotype C: Preserved Brief Intense Tolerance but Poor Sustained Zone 1–2 Tolerance

A clinically paradoxical subgroup may report relative tolerance to very brief intense efforts (stairs, short bursts) yet poor tolerance to sustained low‐intensity walking. Within this model, transient pressure‐mediated perfusion and anaerobic energy pathways may remain relatively preserved, whereas prolonged shear‐dependent endothelial NO signaling remains impaired. Sustained Zone 1–2 activity may require continuous endothelial NO production that the uncoupled eNOS cannot reliably provide, while very brief intense efforts may be sustained via phosphocreatine pathways without the same endothelial demand. Additionally, phosphocreatine‐based energy generation bypasses both the eNOS‐dependent endothelial NO requirement and the Warburg‐mediated ATP deficit, potentially explaining why this subgroup may function comparatively well during maximal brief efforts yet deteriorate with any sustained aerobic demand that depends on oxidative phosphorylation and endothelial NO signaling. This pattern represents a deductive prediction of the model rather than an incidental observation: if eNOS uncoupling specifically impairs sustained NO‐dependent microvascular vasodilation, then activities relying on aerobic oxidative metabolism and continuous endothelial NO production should be disproportionately affected, while brief efforts sustained primarily via phosphocreatine resynthesis which bypasses the eNOS‐dependent endothelial pathway entirely may be relatively spared.

If this mechanism is correct, this subgroup may tolerate intermittent isometric loading better than continuous aerobic sessions. This observation warrants formal physiological investigation and should not be interpreted as evidence of normal cardiorespiratory capacity.

### 4.4 Phenotype D: Progressive Bedbound or Near‐Bedbound State

At the severe end of the spectrum, some individuals may become predominantly housebound or bedbound. Multiple mechanisms may contribute, including severe PEM susceptibility, autonomic dysfunction, immune dysregulation, and deconditioning. The present model adds the possibility that, in some individuals, prolonged reduction in ambulatory shear exposure from the earliest stages of illness may contribute to progressive narrowing of both thresholds not through any failure to attempt activity, but as a potential consequence of the illness itself constraining movement. This is not proposed as a universal pattern, nor as a judgment on those unable to maintain any activity; it is offered as a mechanistic consideration that may apply in a subset of patients and that, if correct, would underscore the importance of even minimal movement where safely possible and under specialist guidance as a means of maintaining residual endothelial signaling rather than as a rehabilitation target.

In this proposed state, the physiotherapy priority may be stabilization rather than exercise prescription: positional management, passive movement if available, very low‐dose isometric contractions to maintain residual KLF2 signaling, and prevention of further circulatory deconditioning. Conventional exercise‐based rehabilitation would be contraindicated until a safe activity window can be empirically established under specialist supervision.

### 4.5 Core Rehabilitation Implication Across Phenotypes

Across all proposed phenotypes, the central implication is that activity intensity alone may be an inadequate guide to safety in this phenotype. Duration, pacing pattern, transition dynamics, and hemodynamic quality of movement are proposed as equally important determinants of endothelial response. If validated, rehabilitation for this phenotype would extend current symptom‐contingent pacing approaches consistent with WHO and NICE guidance by adding a hemodynamic quality dimension: within the individually tolerated pacing envelope, the distinction between sustained laminar and oscillatory shear patterns may be an independent determinant of endothelial outcome. This does not alter the quantity of permitted activity but hypothesizes that the manner in which that activity is performed may matter at the microvascular level. Post‐session root mean square of successive differences (RMSSD) recovery kinetics, resting heart rate trends, and nocturnal HRV (all assessed against the individual's 7–14 day rolling baseline) are proposed as additional exploratory monitoring considerations (discussed and referenced in Sections 7 and 8.1), alongside avoidance of abrupt changes in activity intensity or pace that may generate oscillatory rather than laminar shear patterns; each requires further prospective study before clinical application.

## 5 Proposed Management of the Acute Oscillatory Shear Response

A clinically important distinction with management implications: chest tightness, dyspnea, and palpitations occurring during or immediately after activity may not represent PEM. Within this model, they are proposed as an acute oscillatory shear response in a BH4‐depleted endothelium, consistent with a vasoconstrictive microvascular response and sympathetic amplification. PEM, by contrast, is characterized by onset 12–48 h after activity, multisystem involvement, and prolonged recovery lasting days to weeks.

Misclassifying the acute shear response as PEM may lead to excessive rest prescriptions that further erode the shear stress threshold. Correctly identifying it as an acute hemodynamic event may allow targeted stabilization. Three mechanistically complementary first‐line responses are proposed, none of which require pharmacological intervention.

### 5.1 Immediate Postural Management

Adoption of a supine or semi‐reclined position may reduce cardiac output and blood flow velocity, potentially converting the oscillatory shear pattern generated during exertion back toward a resting laminar state. Lower cardiac output may reduce NADPH oxidase activation and endothelin‐1‐driven microvascular constriction.

### 5.2 Slow Diaphragmatic Breathing

Approximately 6 cycles per minute with extended expiration (4 s inhalation:6–8 s exhalation) may activate parasympathetic tone via the Hering‐Breuer reflex, potentially reducing sympathetic drive and the adrenergic component of vascular tone that may amplify the endothelial inflammatory signal.

### 5.3 Nasal Humming Cycles

Weitzberg and Lundberg [14] demonstrated that humming increases nasal NO output up to 15‐fold compared to quiet exhalation through oscillating airflow enhancing paranasal sinus ventilation. This preformed nasal NO may enter the pulmonary circulation and activate soluble guanylate cyclase in vascular smooth muscle independently of eNOS coupling status, potentially providing vasodilatory support without engaging the uncoupled eNOS pathway. Protocol: 5–10 s nasal humming during exhalation, 30–60 s silence, repeated 5–8 cycles while supine. This approach requires clinical evaluation before recommendation.

These non‐pharmacological responses are proposed for formal evaluation, not clinical implementation without specialist oversight. A counterintuitive but mechanistically important implication of this framework concerns what should follow these acute stabilization responses. Within the proposed model, the appropriate response to acute shear symptoms that have been successfully stabilized and where no delayed multisystem PEM criteria are anticipated to be met is not prolonged immobility. Prolonged rest after a subthreshold exertional episode may withdraw the laminar shear signal required to maintain BH4 synthesis, potentially eroding the shear stress threshold rather than preserving it. The model instead predicts that brief, gentle, continuous low‐velocity movement, slow walking at a pace that generates no acute symptoms, distributed isometric contractions (gluteal, quadriceps, periscapular squeezes of 3–5 s), or sustained nasal humming in the recumbent position may provide sufficient laminar shear stimulus to defend the BH4 pool during the recovery window. This is distinct from attempting to resume the preceding activity: it is the minimum stimulus necessary to prevent further threshold erosion. Oral nitrate intake (beetroot, spinach, pomegranate) during this window may additionally support the enterosalivary NO pathway as a compensatory vasodilatory reserve. A further proposed clinical manifestation of the acute oscillatory shear response and a potential early individual warning signal of impending eNOS uncoupling threshold crossing is a nonproductive throat‐clearing sensation or dry cough arising during exertion in the absence of respiratory pathology. Within the proposed model, as NO bioavailability falls acutely during oscillatory shear, laryngopharyngeal microvascular endothelin‐1‐mediated constriction and mast cell activation may produce a sensation of pharyngeal tightness or retained secretion without productive expectoration. This symptom, if it consistently precedes delayed multisystem worsening in individual patients, may represent a personally calibrated early warning signal that the acute shear response is approaching the proposed PEM threshold distinct from established respiratory disease and resolvable with the postural and breathing interventions described above.

## 6 The Recoupling‐First Principle and Candidate Pathway‐Targeting Interventions: A Framework for Future Trials

NO‐substrate supplementation (L‐arginine, L‐citrulline) in the presence of active eNOS uncoupling may worsen outcomes. This concern derives from the Arginine Paradox documented in the VINTAGE MI trial [15], where L‐arginine supplementation increased postinfarction mortality in a high‐oxidative‐stress setting. If eNOS is actively uncoupled, additional substrate may be converted to peroxynitrite rather than NO. The proposed therapeutic sequence is: stabilize the redox environment first; restore cofactor availability second; consider substrate support only when coupling is confirmed. The following represents a mechanistic mapping of candidate interventions to proposed pathway nodes, offered as a framework for prospective trial design, not as a combined treatment protocol. Each candidate agent requires independent evaluation in Long COVID patients. All agents described are prescription medications in most jurisdictions and require physician evaluation and prescribing.

### 6.1 Layer 1: Inflammation and Oxidative Stabilization

Colchicine may provide NLRP3 inflammasome inhibition and NF‐κB modulation relevant to the proposed inflammatory priming mechanism [16]. Because iNOS expression is NF‐κB‐dependent, colchicine is proposed as the primary agent for closing the iNOS‐driven background BH4 depletion that primes the endothelium for eNOS uncoupling; without suppression of this upstream inflammatory signal, pharmacological correction of eNOS coupling may be biochemically insufficient regardless of cofactor availability. Low‐dose aspirin may reduce thromboxane A2‐mediated vasoconstriction. Na‐R‐alpha‐lipoic acid may serve as a mitochondrial antioxidant. Liposomal Vitamin C may support BH3 radical recycling back to BH4 via the ascorbate‐mediated mechanism described by Kuzkaya et al. [17]: this is proposed as the critical acute‐phase antioxidant intervention because it may slow BH4 depletion without increasing eNOS substrate load. GlyNAC (glycine + N‐acetylcysteine combined) may provide rate‐limiting glutathione precursor support; combined supplementation has been shown to restore glutathione deficiency, oxidative stress, and endothelial dysfunction in post‐viral contexts more effectively than N‐acetylcysteine alone [18]. Omega‐3 fatty acids may modulate NF‐κB signaling. Metformin operates through several mechanisms relevant to the proposed pathway: AMP‐activated protein kinase (AMPK) activation leading to NF‐κB inhibition and reduced iNOS expression, attenuation of mitochondrial Complex I activity reducing ROS generation at source, phosphorylation of eNOS at Ser1177 supporting improved coupling, and NLRP3 inflammasome modulation [19, 20]. Mechanistically, metformin targets the same upstream iNOS‐driving inflammatory signaling as colchicine, and the two agents may represent partially overlapping approaches to the Layer 1 objective of iNOS suppression during the priming phase.

### 6.2 Layer 2: Microvascular Ischemia Protection

Ranolazine acts via late sodium current inhibition, reducing intracellular calcium overload in cardiomyocytes and microvascular smooth muscle under ischemic or metabolically stressed conditions [21]. Its proposed advantage in this phenotype is that it may provide ischemia protection without altering blood pressure or heart rate, thereby avoiding hemodynamic changes that would alter the microvascular shear environment in unpredictable ways in an already‐compromised system.

### 6.3 Layer 3: Endothelial Glycocalyx Restoration

Sulodexide provides glycosaminoglycan precursors for glycocalyx repair and has demonstrated vascular protective properties in clinical studies. The TUN‐EndCOV study reported 83.7% improvement in chest pain and 85.2% improvement in palpitations with 250 LSU twice daily for 21 days in Long COVID patients with proven endothelial dysfunction [22]. Gonzalez‐Ochoa et al. [23] demonstrated significant reduction in thrombomodulin, vWF, D‐dimer, CRP, and IL‐6 with 250 LRU twice daily for 8 weeks in convalescent COVID‐19 patients. These trials do not confirm the eNOS uncoupling mechanism proposed here, but provide supportive clinical data for the glycocalyx restoration approach.

### 6.4 Layer 4: eNOS Recoupling Support

Statin therapy has been shown to upregulate GCH1 gene expression via Rho‐kinase inhibition in human vascular tissue, increasing endothelial BH4 synthetic capacity [24]. A secondary mechanistic benefit is proposed: by maintaining GCH1 expression through Rho‐kinase suppression, statins may additionally counteract the HIF‐1α‐mediated GCH1 suppression described in Section 2.1, providing a dual mechanism for preserving de novo BH4 synthesis capacity even during periods of residual pseudo‐hypoxic signaling. If this mechanism extends to the post‐COVID endothelial context, statin therapy may represent an upstream intervention supporting BH4 resynthesis. Statin therapy may serve as an accessible complement or alternative to sulodexide in settings where availability is limited. The pleiotropic endothelial profile of statins, Rho‐kinase inhibition (upregulating GCH1), NADPH oxidase suppression (via Rac1), and eNOS Ser1177 phosphorylation [25, 26] addresses multiple proposed pathway nodes simultaneously. Observational data suggest that statin use at the time of SARS‐CoV‐2 infection was associated with attenuated disease severity, a signal mechanistically consistent with partial maintenance of eNOS coupling during the acute iNOS‐dominant phase [27]. These findings are hypothesis‐consistent but do not constitute evidence specifically for the eNOS uncoupling phenotype described here; prospective evaluation in this population is required. If statins are evaluated in combination with other candidate agents, pharmacological interaction profiles would require clinical consideration. 5‐methyltetrahydrofolate (5‐MTHF), the active methylated folate form, not synthetic folic acid, may support DHFR‐mediated BH2‐to‐BH4 recycling. Whitsett et al. [28] demonstrated that synthetic folic acid competitively inhibits human endothelial DHFR at concentrations achievable with standard supplementation, a finding that provides mechanistic justification for specifying 5‐MTHF rather than standard folic acid in this framework. If evaluated clinically, sequencing considerations would suggest that cofactor support be explored after initial inflammatory and redox stabilization, given the state‐dependence described in Section 6.

The candidate intervention domains described in this section are presented solely as translational implications arising from the proposed mechanistic hypothesis. They do not constitute treatment recommendations, clinical guidelines, or standards of care. The intervention sequence described reflects mechanistic logic derived from the model and should not be applied clinically without independent prospective evaluation. All agents mentioned are prescription medications requiring physician assessment. Controlled trials specifically targeting this proposed phenotype are required before any clinical application can be considered. The therapeutic candidates described in Sections 6.1, 6.5 are proposed as hypothesis‐generated translational directions for future prospective evaluation, not as clinically validated protocols. The evidence base supporting each candidate derives from established vascular biochemistry applied to the proposed mechanism; none has been evaluated in a controlled trial specifically targeting the eNOS uncoupling phenotype described here. The sequence proposed reflects mechanistic logic, not clinical hierarchy.

### 6.5 Pharmacological and Mechanical Shear Stress Mimetics for Severely Limited Patients

A specific therapeutic challenge arises in severely affected patients (Phenotype D) who are bedbound, near‐bedbound, or otherwise unable to tolerate even constant‐pace low‐intensity ambulation. In this subgroup, the protective laminar shear stimulus required to sustain KLF2‐mediated GCH1 transcription and BH4 synthesis cannot be physiologically generated. Within the proposed model, this represents not merely an exercise limitation but a progressive hemodynamic withdrawal that may further suppress endothelial maintenance pathways and accelerate threshold narrowing. The Two‐Threshold Model predicts that without an alternative shear‐equivalent stimulus, this subgroup may deteriorate even with optimal Layers 1–3 pharmacological support. Two complementary therapeutic categories may address this gap. First, pharmacological shear stress mimetic agents that activate the same KLF2/eNOS signaling cascade triggered by physiological laminar shear have been formally proposed in the COVID‐19 context by Kim and Arany [29]. The proposed mechanism overlaps substantially with the Layer 4 framework (Section 6.4): statins induce KLF2 and GCH1 expression and recouple dysfunctional eNOS in vivo [25, 26]; resveratrol and sulforaphane activate KLF2/Nrf2‐mediated antioxidant transcription; dipyridamole increases endothelial cAMP and supports eNOS function. In the severely limited patient who cannot generate physiological shear, these agents may functionally substitute for the absent mechanical signal at the molecular level. Second, mechanical shear stress mimetic non‐pharmacological interventions that activate the endothelial mechanosensory complex through external means offer an alternative therapeutic axis. Low‐energy extracorporeal shock wave therapy (ESWT) at intensities of 0.012–0.045 mJ/mm^2^ has been shown to activate the identical mechanosensory complex (VEGFR2/VE‐cadherin/PECAM‐1) and induce the same downstream signaling cascade (Akt/eNOS phosphorylation; KLF2, KLF4, eNOS, Nur77 expression) as physiological laminar shear stress [30]. ESWT is already an established physiotherapy modality for musculoskeletal indications and would require minimal infrastructure adaptation for vascular application. Within the proposed framework, low‐energy ESWT applied to peripheral vascular beds may constitute a novel, non‐exertional therapeutic option for the bedbound vascular‐PEM subgroup, requiring no metabolic expenditure and avoiding the oscillatory shear risk inherent to active rehabilitation. The combination of pharmacological and focal mechanical mimetics (ESWT) may offer this subgroup a hemodynamically defensible recovery trajectory previously considered inaccessible. This represents a translational extension of the model rather than an established therapeutic protocol, and would require dedicated controlled evaluation in this specific phenotype. A critical dosing consideration applies: because ESWT acts as a mechanical shear stress mimetic, it may produce hemodynamic effects analogous to those of exercise in this phenotype. Application should therefore begin at the lowest available energy levels, with post‐session monitoring of RMSSD recovery kinetics and resting heart rate the following morning, to ensure that the ESWT stimulus does not cross the individual's proposed PEM threshold. Escalation of ESWT dose should follow the same principle as session dosing in active rehabilitation: contingent on stable post‐session autonomic recovery markers rather than on a fixed schedule. Crucially, the framework predicts that these mimetic interventions should be most effective when paired with concurrent Layers 1–3 support: glycocalyx restoration (sulodexide), inflammatory and redox stabilization, and microvascular protection. Mimetic stimulation of a mechanosensory apparatus that remains structurally and biochemically compromised would be expected to yield diminished returns, consistent with the integrated nature of the cascade described in Section 9.7. Whether this predicted attenuation of response occurs in practice requires direct prospective demonstration and is offered here as a hypothesis‐derived consideration for future study design rather than an established finding.

## 7 Proposed Biomarker Validation Framework

Direct confirmation of this hypothesis requires prospective dynamic measurement before and after a standardized low‐intensity exercise challenge not only at rest. Static baseline measurement is insufficient to test this model, as the proposed signature is a post‐exertional biochemical shift, not a fixed baseline abnormality.

For clinical identification of the vascular endothelial dysfunction phenotype proposed in this hypothesis, post‐occlusive reactive hyperaemia (PORH) protocol with finger thermal monitoring offers an objective, reproducible, and clinically deployable approach. The Endothelium Quality Index (EQI), validated in the TUN‐EndCOV cohort of 290 Long COVID patients [22], provides an operational threshold (EQI < 2.0) for identifying patients with endothelial dysfunction who may be candidates for the therapeutic framework proposed in Sections 5 and 6. This approach allows phenotype‐stratified enrolment in future biomarker validation studies, ensuring that mechanistic conclusions are drawn from patients matching the specific vascular‐PEM presentation rather than from heterogeneous Long COVID populations in which mechanism‐discordant phenotypes may dilute the signal.

### 7.1 Primary Endpoints

BH4/BH2 ratio (proposed to show post‐exertional reduction); nitrotyrosine (proposed to show post‐exertional elevation); asymmetric dimethylarginine (ADMA; proposed to be elevated at baseline and worsen post‐exertion); flow‐mediated dilation (FMD; acknowledged as a validated tool for peripheral conduit artery endothelial function assessment [31], and while FMD interrogates conduit artery endothelial function rather than the microcirculation directly, changes in FMD reflect alterations in eNOS‐derived NO bioavailability mechanistically linked to the proposed cascade, making it a useful systemic surrogate biomarker complementary to the microvascular‐specific PORH protocol described above).

### 7.2 Biomarker Sampling Schedule

Pre‐challenge baseline (fasted, rested); immediately post‐challenge (< 30 min); 24 h post‐challenge; 48 h post‐challenge. The primary analysis window is 24–48 h. Immediate post‐exertional sampling alone would be expected to miss the proposed signature entirely, as the kinetics of DHFR nitration, BH4 depletion, and peroxynitrite accumulation predict a delayed rather than immediate biochemical shift.

### 7.3 Proposed Study Design

Case–control: Long COVID vascular‐PEM phenotype vs. matched healthy controls and vs. recovered Long COVID without PEM. The proposed vascular‐PEM phenotype is not expected to be present in all Long COVID patients with fatigue, but specifically in those with objective evidence of microvascular endothelial dysfunction. Participant selection and the measurement protocol are therefore organized around three sequential levels. Endothelial phenotyping at Level 1 is a prospective inclusion filter, not a diagnostic test for eNOS uncoupling. Participants without objective evidence of endothelial dysfunction at Level 1 may represent alternative biological phenotypes of Long COVID outside the scope of this paper; absence of endothelial dysfunction should not be interpreted as evidence against PEM itself, but as evidence that the participant may belong to a different mechanistic subtype. Level 1. Resting endothelial phenotype: Does the participant have microvascular endothelial dysfunction? The Endothelium Quality Index (EQI), derived from a post‐occlusive reactive hyperaemia (PORH) protocol with finger thermal monitoring and validated in the TUN‐EndCOV cohort of 290 Long COVID patients [22], is applied as the primary phenotype entry filter (EQI < 2.0 = endothelial dysfunction). Two complementary assessments further characterize endothelial function across different vascular domains: (i) FMD (flow‐mediated dilation of the brachial artery) as a surrogate of conduit artery NO‐dependent endothelial function and systemic eNOS‐derived NO bioavailability [31]; (ii) EndoPAT/RHI (peripheral arterial tonometry; RHI ≤ 1.67 = dysfunction) as a validated, FDA‐cleared, operator‐independent measure of digital microvascular reactivity, shown to detect endothelial dysfunction specifically in post‐COVID myalgic encephalomyelitis/chronic fatigue syndrome (ME/CFS) populations [32]. Because no single noninvasive test captures endothelial function across all vascular territories, these complementary assessments reduce phenotypic heterogeneity in the enrolled sample and provide convergent evidence of endothelial dysfunction across resting conditions. Participants without objective endothelial dysfunction on Level 1 assessments are not entered into the mechanistic analysis. This restriction is intentional: the present hypothesis is phenotype‐specific and is designed to investigate the vascular‐endothelial subtype of Long COVID rather than PEM as a universal mechanism. Level 2. Dynamic endothelial function during exercise: Does the resting endothelial dysfunction translate into functional impairment under exertional demand? Participants who pass Level 1 enter the 2‐day CPET protocol (Workwell Foundation protocol [33, 34]): two maximal incremental CPETs on a cycle ergometer, starting at 0 W, administered on consecutive days separated by 24 h. Test termination criteria: RER ≥ 1.1 and RPE ≥ 18/20 (Borg scale), applied identically on both days. The ramp rate is individually selected based on anticipated functional capacity: 10–15 W/min for moderately affected participants, 5–10 W/min for severely affected individuals, and 20–25 W/min for participants with significant prior training history, targeting an 8–12 min test to maximum effort [33]. Primary functional endpoint: reduction in VO2 and workload at the ventilatory anaerobic threshold (VAT) between Day 1 and Day 2; a reduction exceeding normal test–retest variability (> 12% in VO2@VAT) constitutes objective documentation of post‐exertional bioenergetic impairment consistent with PEM [33, 34]. This protocol is recognized in the ACSM Guidelines for Exercise Testing and Prescription as a validated methodology for assessing post‐exertional impairment. NIRS‐derived total hemoglobin (tHb) is monitored continuously via a wearable sensor on the vastus lateralis throughout both CPETs (Day 1 and Day 2). NIRS‐tHb serves as the dynamic physiological readout of exercise‐induced microvascular perfusion, complementing but not replacing the established CPET functional endpoints: it tests whether the resting endothelial dysfunction confirmed at Level 1 translates into measurable microvascular perfusion impairment during exercise a qualitatively different class of information from the resting Level 1 assessments. The mechanistic specificity of NIRS‐tHb derives from the Day 1 to Day 2 comparison rather than from absolute values: healthy deconditioned individuals reproduce tHb response patterns within normal test–retest variability, whereas the proposed uncoupled phenotype is predicted to show progressive microvascular deterioration on Day 2 paralleling the VAT reduction a pattern not explained by deconditioning alone. Specifically: (i) intra‐session tHb may show attenuated rise or paradoxical decline at equivalent workloads on Day 2; (ii) postexercise tHb recovery kinetics (3–5 min post‐cessation) may show attenuated reactive hyperaemia on Day 2. This NIRS signal is interpreted as an exploratory indirect marker of microvascular dynamics, subject to known measurement variability, complementing rather than replacing the established VAT‐based functional endpoints. For participants who do not complete the full CPET on Day 1 or Day 2 due to symptom limitation, the NIRS‐tHb data already collected during the partial test are retained and analyzed. Because the NIRS sensor is worn continuously throughout the test regardless of its duration, even partial CPET sessions yield a within‐session tHb trajectory and a post‐cessation recovery window. The 2‐day CPET protocol with VAT reduction as primary endpoint remains the validated gold standard for documenting PEM [33, 34]; NIRS‐tHb is not proposed as a replacement for this endpoint. However, published evidence supports the presence of abnormal NIRS‐derived muscle oxygenation patterns in ME/CFS and post‐COVID syndrome during exercise: impaired microvascular reperfusion has been documented using NIRS in post‐COVID patients independently of cardiorespiratory fitness [35], and abnormal oxygenated hemoglobin kinetics during submaximal exercise have been reported in ME/CFS compared to healthy controls [36]. On this basis, if Day 1 NIRS‐tHb data show attenuated or paradoxical tHb patterns, and Day 2 data show further deterioration relative to Day 1 in a participant who also shows endothelial dysfunction at Level 1 this convergent pattern is retained as exploratory evidence consistent with the proposed phenotype, pending biomarker confirmation at Level 3. Biomarker sampling proceeds on the same schedule regardless of whether the CPET was completed. Participants who do not complete either CPET session are not excluded; their data are retained for prespecified exploratory subgroup analysis. Level 3. Mechanistic discrimination: Is the endothelial dysfunction mediated by eNOS uncoupling or an alternative oxidative pathway? Biomarker sampling: BH4/BH2 ratio, nitrotyrosine, asymmetric dimethylarginine (ADMA an endogenous eNOS inhibitor that competes with L‐arginine for the eNOS active site, providing an independent index of eNOS substrate availability), GSH/GSSG, and 8‐isoprostane sampled pre‐exercise baseline, immediately postexercise (< 30 min), 24 h, and 48 h postexercise on both Day 1 and Day 2. The primary analysis window is 24–48 h post‐exertion, as the proposed kinetics of DHFR nitration, BH4 depletion, and peroxynitrite accumulation predict a delayed rather than immediate biochemical shift; static baseline measurement alone is insufficient to test this model. Only the biochemical biomarker level provides mechanistic specificity for eNOS uncoupling versus alternative oxidative pathways. The protocol is self‐stratifying: (i) if BH4/BH2 ratio declines and nitrotyrosine rises post‐exertion in a participant with confirmed PEM and confirmed endothelial dysfunction eNOS uncoupling is specifically implicated; (ii) if GSH/GSSG and 8‐isoprostane are elevated but BH4/BH2 ratio is preserved alternative oxidative or non‐eNOS mechanisms including but not limited to those discussed in Section 9.8 are more likely as the primary driver in that participant, though nonspecific elevation of oxidative stress markers following exertion cannot be excluded without additional mechanistic evidence. The protocol is designed to test whether eNOS uncoupling is the primary driver in this phenotype; it is not designed to identify which alternative mechanism predominates when it is not. Discriminating among alternative oxidative and non‐eNOS pathways would require additional, pathway‐specific measurements. The resulting dataset would nonetheless remain informative if the primary hypothesis were not supported: because standardized phenotyping, objective post‐exertional bioenergetic measurement, microvascular assessment, and a panel of redox and endothelial markers are obtained in the same participants, the results would narrow the range of mechanisms compatible with this phenotype and provide a characterized cohort and reference dataset against which alternative models, including those outlined in Section 9.8, could be evaluated in subsequent work. Prespecified genetic covariate: MTHFR C677T is included as a prespecified exploratory stratification covariate. Its inclusion is motivated by its established role in folate‐dependent one‐carbon metabolism and potential indirect effects on BH4 recycling capacity, which may modulate individual susceptibility to BH4 depletion under oxidative challenge. It is not modeled as a causal component of the primary eNOS uncoupling pathway, and its predictive value in this phenotype requires prospective evaluation. Falsification prediction: In participants meeting Level 1 endothelial dysfunction criteria and demonstrating post‐exertional bioenergetic impairment on the 2‐day CPET protocol (VAT reduction > 12% on Day 2 relative to Day 1), the eNOS uncoupling mechanism predicts two primary, mechanism‐specific biochemical signals: (i) a post‐exertional decline in the BH4/BH2 ratio; and (ii) a post‐exertional rise in nitrotyrosine. An attenuated or paradoxical NIRS‐tHb response on Day 2 relative to Day 1 is treated as a supportive physiological observation rather than a primary criterion, as it lacks mechanistic specificity. Accordingly, the mechanism is supported if both primary signals are present, with or without the supportive microvascular finding; it is not supported as the primary driver in this phenotype if both primary signals are absent despite confirmed PEM and confirmed endothelial dysfunction, irrespective of the NIRS‐tHb result; and a discordant result, in which only one primary signal is present, is considered indeterminate and would require further mechanistic investigation. This falsifiability is a deliberate design feature of the model.

## 8 Implications for Physiotherapy Practice

All guidance in this section is presented as a hypothesis‐derived framework requiring prospective clinical evaluation under specialist supervision before implementation; it does not constitute a clinical protocol. Current WHO [37] and NICE [38] guidelines recommend pacing rather than graded exercise therapy for patients with post‐exertional symptom exacerbation. The Two‐Threshold Model is fully consistent with this guidance, adding a hemodynamic quality dimension within the existing pacing envelope without altering its fundamental principle. Three clinically important clarifications follow from the proposed model. First, symptoms occurring during or immediately after gentle activity, transient tachycardia, mild breathlessness, slight fatigue do not necessarily indicate that the PEM threshold has been crossed. Within the proposed model, these may reflect an acute autonomic compensation response to peripheral vasodilatory insufficiency, not a threshold‐crossing event. Misclassifying this acute response as PEM may lead to progressive reduction of all activity, withdrawing the laminar shear stimulus required for KLF2‐mediated GCH1 transcription and BH4 synthesis, and potentially accelerating BH4 pool depletion even without any exertional trigger. Second, heart rate during activity is proposed to be an insufficient safety guide in this phenotype. Intra‐session tachycardia may reflect compensatory sympathetic activation secondary to endothelial dysfunction; the endothelium attempts vasodilation, but the NO‐mediated peripheral resistance reduction is insufficient, prompting compensatory cardiac output increase rather than a signal of excessive metabolic demand or threshold crossing, and terminating activity based on heart rate alone may deprive the patient of the laminar shear stimulus the session was intended to provide. The relevant monitoring parameters are proposed to be post‐session rather than intra‐session. The primary proposed monitoring parameter is RMSSD recovery kinetics: the time for RMSSD to return to presession baseline, which across stable sessions establishes the individual T_ind reference (described in Section 8.1). Preliminary evidence from Long COVID populations is consistent with this approach, showing that HRV remains suppressed for up to 24 h after exercise at intensities approaching individual exertional thresholds, while subthreshold exercise is associated with more normal recovery trajectories [39]. Third, complete immobility is not mechanistically neutral in this phenotype. The model predicts that prolonged rest may erode the shear stress tolerance threshold by withdrawing the principal physiological maintenance signal for endothelial BH4 synthesis—a risk that applies both post‐exertion and during extended sedentary periods independently of exercise.

The proposed limitation of applying activity programs that focus exclusively on symptom‐contingent dose limits, without accounting for hemodynamic quality, is that such approaches target the PEM threshold as the primary safety boundary. Within this model, this may be necessary but insufficient. A program avoiding PEM events may still fail to maintain the laminar shear stimulus proposed to sustain BH4 synthesis, particularly if prescribed activities generate oscillatory rather than laminar shear even at sub‐PEM intensity levels. The predicted consequence would be a program that remains symptomatically safe while progressively eroding endothelial BH4 reserve, eventually producing deterioration that appears paradoxically to arise without obvious overexertion. This prediction requires prospective testing and is offered as a falsifiable hypothesis, not a clinical claim.

Five operational implications are proposed, each requiring clinical validation before adoption:

1. The proposed safety target is both thresholds simultaneously: the PEM threshold should not be crossed, and the shear stress threshold should not fall chronically below the proposed BH4 maintenance minimum.
2. Within this model, activity modality may matter independently of intensity: constant‐pace walking may generate protective laminar shear (10–20 dyn/cm^2^) while variable‐pace, interval, or stop‐start patterns may generate oscillatory shear that is potentially counterproductive even at low average HR values [10, 13].
3. Heart rate may be insufficient as the sole monitoring metric for this phenotype. The within‐session stability of the respiratory rate during activity is proposed as a more accessible noninvasive indicator: a disproportionate rise in respiratory rate relative to walking pace may signal proximity to oscillatory shear territory, prompting pace reduction; if stabilization is not achievable, the session should be concluded. This approach requires prospective validation.
4. The proposed therapeutic goal is shear threshold maintenance and progressive shear threshold restoration, not conventional fitness progression. Success would be measured as expanding safe session duration without post‐exertional response, not increasing intensity.
5. The entry dose for severely deconditioned patients should be calibrated against the individual's empirically established safe ceiling, not normative aerobic capacity. This may require starting at very low doses (3–5 min of constant‐pace walking) in some patients.

### 8.1 Phenotype‐Stratified Application of the Two‐Threshold Model

The central insight across all functional phenotypes is identical: the capacity to gain new shear stress thresholds depends on preserving the laminar shear–BH4 buffer pathway. When this buffer is lost through sustained inactivity or repeated oscillatory shear exposure consistent with experimental evidence that low or absent laminar shear directly induces eNOS uncoupling [11, 40] the patient loses not only current tolerance but the biochemical precondition for future improvement. Phenotype‐specific guidance is therefore organized around this same principle, applied at different positions on the threshold map.

A mechanistic implication of the proposed cascade warrants explicit acknowledgement before phenotype‐specific guidance is presented. The Two‐Threshold Model predicts as a falsifiable hypothesis rather than a clinical recommendation that physiotherapy protocols applied in isolation, without concurrent attention to the proposed biochemical substrate, will produce attenuating returns over time. Within the proposed model, each session approaching the PEM threshold deposits cumulative DHFR nitration damage regardless of whether PEM is triggered. Without concurrent reduction in iNOS‐driven oxidative load (proposed Layer 1), glycocalyx restoration (proposed Layer 3), and eNOS recoupling support (proposed Layer 4), the BH4 pool available for laminar shear‐mediated resynthesis may progressively contract even in sessions that appear symptomatically safe. The model therefore generates the specific, testable prediction that the same physiotherapy protocol will produce superior and sustained threshold expansion when applied concurrently with Layers 1–4 biochemical support compared with physiotherapy alone, a prediction evaluable in a prospective research design using BH4/BH2 ratio (as a laboratory‐measured mechanistic endpoint) and RMSSD recovery kinetics (as a clinically accessible exploratory monitoring parameter) as complementary outcome markers. This mechanistic prediction is offered as a hypothesis‐derived explanation for a clinical pattern that has been anecdotally noted in this phenotype, namely, that exercise‐based rehabilitation attempted without concurrent attention to the proposed biochemical substrate may produce initial apparent improvement followed by relapse; though this observation has not been prospectively validated and is presented here solely as a falsifiable prediction of the model, not as an established clinical finding.

#### 8.1.1 Bedbound and Severely Limited Patients

Exercise‐based rehabilitation is contraindicated until biochemical stabilization through Layers 1–4 is established. The physiotherapy priority is maintenance of residual hemodynamic signaling through brief isometric contractions distributed throughout the day: gluteal, quadriceps, and periscapular squeezes of 3–7 s every 2–3 h. These generate reactive hyperaemia upon release, providing episodic laminar shear bursts that may preserve residual GTPCH‐1 signaling without metabolic expenditure or oscillatory flow exposure. Nasal humming is of particular importance in this subgroup: providing sustained oscillatory airflow through paranasal sinuses, it generates NO independently of eNOS coupling status and may offer a hemodynamically significant vasodilatory stimulus accessible even to the most severely limited patients, requiring no physical exertion. Regular humming cycles 5–10 s per exhalation, repeated for 5–8 cycles, are proposed as a low‐cost adjunct to isometric contraction protocols in this population. Oral microbiome integrity requires specific attention: the enterosalivary NO pathway depends on nitrate‐reducing bacteria (*Rothia* and Neisseria spp.) resident on the tongue dorsum, which are rapidly depleted by antiseptic mouthwashes and in states of oral dysbiosis. Chronic halitosis may serve as a clinical indicator of preexisting oral dysbiosis compromising this compensatory NO reserve. Probiotic oral supplementation with *Lactobacillus salivarius* K12 (BLIS K12) may support restoration of a NO‐compatible oral microbiome, though this application requires clinical evaluation. Dietary nitrate (beetroot, spinach, arugula, pomegranate) and antioxidant support provide partial non‐eNOS NO compensation while eNOS recoupling is pursued through the pharmacological framework. L‐arginine and L‐citrulline should be avoided: in the presence of uncoupled eNOS, these substrates may increase superoxide and peroxynitrite generation rather than provide NO. Progression to short constant‐pace walking is considered only once a measurable safe activity window—any duration of ambulation producing no acute oscillatory shear response and no delayed multisystem worsening at 48 h has been empirically identified.

#### 8.1.2 Sprint‐Tolerant but Zone 1–2‐Intolerant Patients

This phenotype carries a specific clinical risk: preserved tolerance for brief intense efforts may mislead both patient and clinician into believing the cardiovascular system is functioning adequately, when in fact the laminar shear–BH4 pathway is severely impaired. Within this model, interval and sprint‐pattern activity regardless of average heart rate generates oscillatory shear in microvascular bifurcations, suppresses GTPCH‐1 phosphorylation, and accelerates BH4 depletion. Continued interval training in this phenotype may therefore progressively lower the shear stress threshold despite apparent session‐level tolerance. The recommended approach is complete cessation of all interval and variable‐pace activity. Layers 1–4 are initiated concurrently. Rehabilitation begins exclusively with distributed isometric protocols for an initial period, before carefully trialing short constant‐pace walking at the lowest identifiable safe ceiling. The isometric‐to‐continuous aerobic progression is governed by the appearance of a symptom‐free laminar shear window, not a fixed time schedule.

#### 8.1.3 Patients Tolerating Sustained Zone 1–2 Activity

These patients occupy the most therapeutically favorable position: the laminar shear–BH4 pathway is partially functional, providing a biochemical substrate for threshold rebuilding. Two concurrent objectives are pursued. First, Layers 1–4 support is maintained to progressively improve eNOS coupling capacity, increasing the BH4 yield each laminar shear stimulus produces. Second, consistent constant‐pace sessions at the established safe ceiling are performed at regular intervals to continuously refill the BH4 pool. Threshold testing biomarker‐gated cautious probing of session duration at unchanged pace, not systematic escalation is introduced only during stable periods with no PEM events and stable HRV for at least 1 week, and only when RMSSD recovery has returned within T_ind for at least three consecutive sessions. This approach is mechanistically distinct from GET: escalation is contingent on individual physiological response, not scheduled, and the total activity envelope does not exceed the individual's pacing‐defined symptom ceiling. When acute oscillatory shear symptoms appear during or immediately after a session (chest tightness or dyspnea resolving within 1–2 h without delayed multisystem features), this marks the current shear ceiling without constituting a PEM threshold crossing. The post‐session 24‐h window is then actively managed: antioxidant support, oral nitrate intake via the enterosalivary pathway [41], and gentle distributed movement are prioritized to defend the BH4 pool before its depletion can approach the PEM threshold. Each session successfully completed within the safe window without delayed worsening represents one increment of threshold expansion, the cumulative signal that a new, higher shear stress tolerance threshold has been established. To support objective session dosing without relying on fixed workload targets, a personalized RMSSD recovery index (T_ind) is proposed as a practical titration tool for physiotherapists and patients. Before each session, resting RMSSD is recorded over a 3‐min period of seated rest (presession baseline). The session is then performed. Immediately after completion, the participant returns to seated rest and the time required for RMSSD to return to the presession baseline level is measured. Across stable sessions at a consistent dose, this recovery time establishes the individual's T_ind, a personalized reference that reflects their current autonomic buffering capacity at that training load. Session dosing is governed by comparing post‐session RMSSD recovery kinetics against this baseline: sessions returning to presession RMSSD within T_ind support dose maintenance or cautious progression; sessions requiring up to 1.5 × T_ind are considered borderline and warrant unchanged dosing, under physiotherapist review; sessions in which RMSSD fails to recover within 1.5 × T_ind indicate likely threshold proximity and should prompt reduction of the subsequent session duration or pace [42]. This translates the Two‐Threshold Model into a device‐derived, individually calibrated monitoring signal accessible via consumer‐grade HRV. RMSSD recovery kinetics remains exploratory and requires prospective validation; preliminary evidence shows HRV suppressed for up to 24 h after exercise approaching individual exertional thresholds in Long COVID [39].

### 8.2 The Post‐Exertional Recovery Window as a Proposed Modifiable Phase

Within the proposed framework, PEM severity may depend not only on the initiating exertional load, but also on the biochemical and hemodynamic environment during the subsequent 24–48 h recovery period. If post‐exertional oxidative stress remains elevated, endothelial NO bioavailability falls, and prolonged inactivity reduces laminar shear signaling, a transient exertional insult may progress into a self‐amplifying nitro‐oxidative cascade.

This raises the possibility that in a subset of patients, complete post‐exertional immobility may not always be hemodynamically neutral. If ambulatory shear is the primary maintenance signal for endothelial BH4 synthesis, its abrupt removal after exercise may theoretically withdraw one mechanism available to modulate the post‐exertional oxidative environment. Conversely, recovery states characterized by preserved gentle movement, maintained physiological flow patterns, and antioxidant sufficiency may theoretically support more favorable post‐exertional biochemistry.

Recovery‐period strategies including dietary nitrate intake [41] and antioxidant support for BH4 protection may warrant investigation as adjuncts to post‐exertional management. In practical terms, this does not mean continuous walking after exercise; rather, it means avoiding immediate and prolonged immobility. Distributed gentle movement throughout the recovery day, simple household tasks, brief slow walks where tolerated, or even seated isometric contractions (gluteal, quadriceps, abdominal squeezes of 6–7 s) may be sufficient to maintain residual laminar shear signaling without exceeding the pacing envelope. These strategies remain within the symptom‐contingent activity framework recommended by WHO and NICE and do not constitute an instruction to exercise. Relevant post‐session monitoring parameters are RMSSD recovery kinetics, resting heart rate trends, and nocturnal HRV against the 7–14 day rolling baseline rather than intra‐session heart rate. These concepts remain hypothetical and should not be interpreted as treatment recommendations without prospective clinical testing.

If validated, this concept would suggest that PEM risk reflects not only the exertional stimulus, but also the quality of the recovery state that follows a potentially important reframing for rehabilitation design.

## 9 Discussion

### 9.1 What This Model Proposes Beyond Documented Endothelial Dysfunction

While endothelial dysfunction, oxidative stress, and microvascular abnormalities are now documented in Long COVID [1, 43, 44, 45, 46], the present model attempts to extend these observations by proposing a specific, dynamic, and testable biochemical mechanism. Current literature documents these findings as largely descriptive and static. Current pacing guidance does not specify the hemodynamic quality of permitted activity. Within the proposed model, pacing that maintains laminar shear is mechanistically distinct from pacing that generates oscillatory shear at the same symptom intensity. Furthermore, complete rest after a subthreshold exertional event may paradoxically erode the shear stress tolerance threshold by withdrawing the principal physiological maintenance signal for endothelial BH4 synthesis. Gentle, continuous, low‐velocity movement precisely below the oscillatory shear threshold is proposed as more protective than immobility during the recovery window. This represents a mechanistically grounded extension of, rather than challenge to, current pacing‐based management approaches and warrants prospective investigation. The present model adds several novel elements, each of which generates independently testable predictions: (1) eNOS uncoupling as a proposed central functional mechanism rather than a secondary consequence; (2) dynamic BH4/BH2 imbalance specifically proposed to worsen in the post‐exertional state; (3) a proposed state‐dependent shift from NO signaling to peroxynitrite‐mediated oxidative injury; (4) a proposed self‐amplifying loop in which each mobilization event may further lower the threshold for subsequent dysfunction; (5) the Two‐Threshold Model as a proposed operational model requiring separate management of PEM and shear stress thresholds; and (6) the proposal that activity hemodynamic quality may be an independent determinant of endothelial response, independent of metabolic intensity.

### 9.2 The Enterosalivary NO Pathway as a Proposed Compensatory System

In the context of proposed eNOS uncoupling, the enterosalivary nitrate‐nitrite‐NO pathway may provide a partially eNOS‐independent NO source [41]. This pathway depends on oral microbiome‐mediated reduction of dietary nitrate by nitrate‐reducing bacteria (*Rothia* and *Neisseria* spp.) resident on the tongue dorsum. Impairment of this pathway through oral dysbiosis, antiseptic mouthwash use, or reduced dietary nitrate intake may create a dual‐failure scenario in which both endothelial eNOS and its microbiome‐dependent compensatory counterpart are simultaneously impaired, potentially amplifying the net NO deficit and contributing to interindividual variation in phenotype severity.

### 9.3 Genetic Modifiers

Interindividual susceptibility may be partially influenced by genetic variability in antioxidant defense systems. Katsarou et al. [47] identified polymorphisms in antioxidant‐related genes as potential modifiers of Long COVID risk even following mild infection. Within the proposed framework, impaired redox resilience may lower the threshold for BH4 oxidation and facilitate earlier transition toward eNOS uncoupling under inflammatory conditions. MTHFR C677T is included as a prespecified exploratory stratification variable in future studies, as reduced DHFR activity may amplify BH4 recycling impairment. More broadly, any condition reducing BH4/BH2 ratio below its critical threshold prior to inflammatory trigger may lower the barrier to the proposed cascade. Table 1 presents a proposed taxonomy of predisposing factors organized by mechanism, offered as a testable extension of the model and not as a clinical risk classification. Table 2 presents corresponding protective factors, each mechanistically derived from the same framework. The findings of Katsarou et al. [47] associating SOD2, EPHX1 polymorphisms and preexisting thyroid disease with Long COVID susceptibility and the GRECCO‐19 trial finding that colchicine reduced D‐dimer increase and clinical deterioration during acute COVID‐19 [48] provide independent empirical signals consistent with the predictions of both tables, though neither study was designed to test the specific eNOS uncoupling hypothesis proposed here. The COVID‐OUT randomized controlled trial [19] provides a particularly informative temporal signal. In 1125 participants followed over 10 months, early metformin administration during acute SARS‐CoV‐2 infection reduced Long COVID incidence by 41% (HR 0.58, 95% CI 0.38–0.88, *p* = 0.009); when started within 4 days of symptom onset, the hazard ratio fell to 0.37. Within the proposed model, metformin's mechanistic profile AMPK‐mediated NF‐κB inhibition, iNOS suppression, reduced mitochondrial Complex I‐derived ROS, and improved eNOS Ser1177 phosphorylation is particularly well‐matched to interruption of the iNOS‐driven priming phase described in Section 2.1. This interpretation is further supported by a complementary observation: when metformin has been evaluated in patients with already‐established Long COVID, clinical benefit has been substantially less consistent, with a recent survey reporting no improvement in over half of respondents. This temporal pattern strong preventive effect during the acute iNOS‐dominant phase, modest or absent effect once eNOS uncoupling is established is specifically predicted by the present model, which proposes that eNOS recoupling in the established phase requires combined redox stabilization, DHFR cofactor support, and restoration of laminar shear signaling rather than upstream inflammatory suppression alone. Whether this interpretation is correct requires direct prospective testing; it is offered here as a hypothesis‐derived reading of otherwise paradoxical clinical data. Two recent randomized controlled trials are consistent with this mechanistic specificity argument. The STIMULATE‐ICP consortium randomized 778 adults with Long COVID to colchicine or comparators across 12 UK NHS clinics: colchicine produced a small but statistically significant short‐term fatigue reduction, but the effect was not sustained after drug withdrawal, and the authors concluded that long‐term benefit is unlikely with these drugs alone [49]. A double‐blind placebo‐controlled RCT by Bassi et al. [50] (George Institute for Global Health India) found that colchicine produced no improvement over placebo in functional capacity, respiratory function, or inflammatory markers at 52 weeks in 346 Long COVID patients. Within the proposed model, these results are mechanistically expected: colchicine addresses the iNOS‐driven inflammatory component (Layer 1) but cannot restore eNOS coupling or laminar shear signaling in isolation. The modest transient benefit in STIMULATE‐ICP is consistent with partial upstream suppression of BH4 depletion, while the null functional result of Bassi et al. is consistent with the model's prediction that colchicine is necessary but not sufficient therapeutic benefit requires concurrent eNOS recoupling, BH4 stabilization, and hemodynamic quality of activity. One possible interpretation, consistent with the present model, is that these trials enrolled biologically heterogeneous Long COVID populations rather than a phenotype enriched for endothelial dysfunction; if so, the mechanistically relevant subgroup may have been diluted within the broader sample. This interpretation requires prospective testing and does not preclude alternative explanations, including the possibility that the proposed mechanism is not operative in this condition.

**TABLE 1:** Proposed taxonomy of predisposing factors that may lower the BH4/BH2 ratio below the critical eNOS uncoupling threshold prior to or during an inflammatory trigger, derived from the mechanistic model. All entries represent hypothesis‐derived predictions contingent on the validity of the proposed eNOS uncoupling cascade; none constitutes an established clinical risk classification. Data for SOD2/EPHX1/thyroid findings are derived from Katsarou et al. [47].

Factor category	Specific factors	Proposed mechanism (if model is valid)
Genetic BH4 synthesis	GCH1 polymorphisms	Reduced GTPCH‐1 activity → lower baseline BH4 de novo synthesis
	MTHFR C677T/A1298C	Reduced DHFR cofactor availability → impaired BH2 → BH4 recycling
	eNOS Glu298Asp, T‐786C, intron 4 VNTR	Reduced eNOS expression or BH4 affinity → lower tolerance to BH4/BH2 imbalance
Genetic redox defense	SOD2, GPx1, catalase variants	Impaired ROS clearance → accelerated BH4 oxidation per inflammatory event [47]
	EPHX1 polymorphisms	Reduced microsomal epoxide hydrolase activity → impaired clearance of oxidative metabolites → accelerated BH4 oxidation; associated with Long COVID progression [47]
	NRF2 pathway variants	Reduced antioxidant gene upregulation under oxidative load
Metabolic/comorbid	Type 2 diabetes/hyperglycemia	AGE products → ONOO^−^‐mediated BH4 oxidation; ADMA accumulation → eNOS inhibition
	Hypertension	Angiotensin II → RhoA/Rho‐kinase → GCH1 suppression → reduced BH4 synthesis
	Obesity/metabolic syndrome	Chronic NF‐κB activation → baseline iNOS tone → background BH4 depletion
	Chronic kidney disease	ADMA accumulation → eNOS competitive inhibition → uncoupling‐prone state
	Autoimmune conditions	Persistent NF‐κB activation → chronically elevated iNOS baseline
	Preexisting thyroid disease	Thyroid hormone dysregulation modifies eNOS expression and coupling efficiency; identified as significant aggravating factor in Long COVID cohort [47]
Nutritional/lifestyle	Low dietary nitrate intake	Impaired enterosalivary NO pathway → reduced compensatory NO reserve
	Antiseptic mouthwash use	Elimination of nitrate‐reducing tongue microbiome → loss of enterosalivary pathway
	Low Vitamin C/glutathione	Impaired BH3 → BH4 ascorbate recycling; reduced ROS buffering capacity
	Elevated ferritin/labile iron	Fenton reaction primed → hydroxyl radical availability for BH4 oxidation
	Chronic psychological stress	Cortisol → NF‐κB → baseline iNOS elevation → background BH4 depletion
	High‐volume endurance training pre‐illness	Upregulated eNOS density → proportionally greater superoxide generation if uncoupled
Inflammatory triggers	SARS‐CoV‐2 infection	Index trigger in this model: NF‐κB → iNOS → ONOO^−^ → BH4 depletion + DHFR nitration
	Other viral infections (influenza, EBV, enterovirus)	Same NF‐κB/iNOS cascade; severity depends on infectious burden and host redox reserve
	Repeated or prolonged NF‐κB‐activating stimuli (any source)	Cumulative iNOS activation episodes may progressively deplete BH4 reserve below critical threshold between acute events; specific trigger category less relevant than aggregate oxidative burden
	Major surgery/trauma	Systemic inflammatory response → iNOS activation → BH4 consumption

**TABLE 2:** Proposed protective factors that may maintain the BH4/BH2 ratio above the critical eNOS uncoupling threshold during and following an inflammatory insult, derived from the mechanistic model. All entries represent hypothesis‐derived predictions contingent on the validity of the proposed cascade. Pharmacological entries require physician evaluation and prescribing; none constitutes a treatment recommendation. Intermittent fasting entries refer to moderate time‐restricted eating (16:8 or 14:10) and are not recommended during active post‐exertional malaise episodes [48].

Protective domain	Specific intervention/factor	Proposed mechanism of BH4/eNOS protection (if model is valid)
Dietary nitrate	Beetroot, spinach, arugula, leafy greens daily	Enterosalivary NO pathway → eNOS‐independent NO reserve; compensates for eNOS coupling deficit during and after inflammatory insult
	Preserve oral nitrate‐reducing microbiome (avoid antiseptic mouthwash)	Maintains *Rothia*/*Neisseria* spp. on tongue dorsum required for nitrate → nitrite → NO conversion [41]
	Presession inorganic nitrate (NO_3_‐T/betaine nitrate, beetroot concentrate, arugula, spinach) 60–90 min before physiotherapy or structured movement sessions	Enterosalivary NO pathway activation providing eNOS‐independent vasodilatory reserve; proposed to attenuate acute oscillatory shear response and support post‐session BH4 defense window; requires intact oral nitrate‐reducing microbiome
Antioxidant nutrition	Vitamin C (dietary or liposomal supplementation)	BH3 → BH4 ascorbate‐mediated recycling; slows BH4 oxidation rate without increasing eNOS substrate load [17]
	Polyphenols/flavonoids (Mediterranean diet, berries, green tea)	ROS buffering → reduced BH4 oxidation rate; NF‐κB modulation → reduced baseline iNOS tone
	Omega‐3 fatty acids (oily fish, flaxseed)	NF‐κB/NLRP3 modulation → reduced iNOS expression; antithrombotic effects on microvascular beds
	Glutathione precursors (NAC, whey protein)	Glutathione reserve → ROS buffering; protects DHFR from peroxynitrite nitration
	Na‐R‐ALA (stabilized R‐alpha lipoic acid)	Mitochondrial ROS scavenging; regenerates Vit C and glutathione; supports DHFR protection from peroxynitrite nitration
	GlyNAC (glycine + N‐acetylcysteine combined)	Rate‐limiting GSH precursors; combined supplementation restores glutathione depletion in post‐viral contexts; supports antioxidant defense of BH4 pool
Activity and sleep	Consistent Zone 1–2 aerobic activity pre‐illness	Chronic laminar shear → KLF2 → GCH1 → high baseline BH4 reserve; larger buffer before critical uncoupling threshold
	Adequate sleep (7–9 h)	NF‐κB suppression during sleep → reduced baseline iNOS tone; HRV and autonomic recovery
	Intermittent fasting (16:8 or 14:10 pattern) not during active PEM	AMPK activation → NF‐κB inhibition → reduced iNOS expression; autophagy → clearance of nitrated DHFR; SIRT1 → eNOS deacetylation → improved coupling efficiency
Stress management	Psychological stress reduction (mindfulness, sleep hygiene)	Cortisol reduction → attenuated NF‐κB → lower baseline iNOS tone → slower background BH4 depletion
	Avoidance of smoking and high air pollution exposure	Reduced exogenous oxidative burden on BH4 pool
Early pharmacological (physician supervised)	Colchicine during acute infection	NLRP3 inflammasome/NF‐κB inhibition → reduced iNOS induction; GRECCO‐19 RCT demonstrated smaller D‐dimer increase and reduced clinical deterioration rate (1.8% vs. 14.0%, NNT 7.1) [48]
	Metformin during acute infection	AMPK → NF‐κB inhibition → reduced iNOS; reduced mitochondrial Complex I ROS; eNOS Ser1177 phosphorylation; COVID‐OUT RCT demonstrated 41% reduction in Long COVID incidence (HR 0.58; 0.37 if started < 4 days) [19]
	Statin therapy (low‐dose rosuvastatin or equivalent, physician supervised)	GCH1 upregulation via Rho‐kinase inhibition → ↑ BH4 synthesis; NADPH oxidase suppression via Rac1 inhibition → ↓ superoxide; eNOS Ser1177 phosphorylation → ↑ coupling; direct eNOS recoupling demonstrated in vivo [25, 26]; broadly accessible with extensive safety profile
	ACE inhibitors/ARBs	Angiotensin II blockade → attenuated RhoA/Rho‐kinase → partial GCH1 preservation → maintained BH4 synthesis during acute inflammatory phase
	Liposomal Vitamin C (high dose during acute phase)	Rapid BH3 → BH4 recycling during acute oxidative surge; reduces rate of BH4 pool depletion before DHFR nitration accumulates [17]
Cofactor support (physician supervised)	5‐MTHF (not synthetic folic acid) post‐acute phase	DHFR cofactor support → BH2 → BH4 recycling; synthetic folic acid competitively inhibits DHFR at achievable supplementation doses [10]

### 9.4 Scope, Limitations, and Boundary Conditions of the Proposed Framework

This hypothesis is phenotype‐specific and does not propose a universal mechanism for Long COVID or ME/CFS. The proposed mechanism does not claim exclusivity: eNOS uncoupling is presented as a hierarchically central node within a broader endothelial dysfunction network, whose additional components including EDH impairment, mitochondrial dysfunction, and glycocalyx injury are addressed in Section 9.8. The proposed phenotype is characterized by: (1) PEM with 12–48 h delayed onset; (2) chest tightness and/or dyspnea appearing at rest, not during activity; (3) normal macrovascular cardiac testing; (4) suspected microvascular dysfunction; and (5) suppressed HRV below personal baseline after minimal activity.

It is important to emphasize that the proposed phenotype is characterized by continuous impairment throughout the entire waking day, not only by responses to exertion. The resting dyspnea, cognitive impairment, and persistent fatigue that characterize this phenotype during periods of inactivity reflect the iNOS‐mediated pseudo‐hypoxia and Warburg‐mediated energy deficit described in Section 2.1, which operate independently of exertional shear stress. The four functional phenotypes described in Section 4 each represent a response pattern layered upon this continuous baseline impairment. Any study design or rehabilitation framework that evaluates outcomes only during or after exercise will fail to capture the full burden of this phenotype, and any biomarker protocol that measures only post‐exertional states will miss the resting biochemical abnormalities that this model predicts. Specific language in the physiotherapy section reflects predictions of the proposed model, not clinical guidelines. Direct in vivo demonstration of eNOS uncoupling in Long COVID patients has not yet been performed; the proposed mechanism is inferred from established vascular biochemistry applied to the Long COVID context. DHFR nitration by peroxynitrite in this specific condition remains to be prospectively demonstrated. These limitations are acknowledged as central to the hypothesis framing of this paper.

### 9.5 Relationship to Competing Mechanistic Models

Multiple mechanistic models have been proposed for Long COVID PEM, and the present hypothesis is not offered as a replacement for these frameworks but as a potentially complementary mechanistic pathway that may be primary in a specific vascular‐endothelial subtype. Mitochondrial dysfunction, including impaired oxidative phosphorylation and Complex I activity, has been documented in post‐exertional skeletal muscle biopsies [44] and may represent a parallel or downstream contributor to PEM in phenotypes where metabolic threshold impairment is primary rather than perfusion limited. Fibrin microclot formation and impaired fibrinolysis, described by Pretorius et al. in Long COVID, may produce capillary occlusion that amplifies the tissue hypoperfusion proposed in our model, potentially interacting with eNOS uncoupling at the microvascular level. Neuroimmune mechanisms including mast cell activation, neuroinflammation, and persistent viral reservoirs represent additional candidate pathways that may be primary in nonvascular PEM phenotypes. The present model does not compete with these frameworks: rather, it proposes that in patients presenting with the specific vascular‐PEM phenotype described in Section 2, endothelial redox switching may be the upstream event from which autonomic, metabolic, and perfusion abnormalities partially derive. The eNOS‐centered framework proposed here is further contextualized by emerging evidence in ME/CFS, a closely related condition: Bertinat et al. [51] demonstrated, in a controlled in vitro study, that plasma from ME/CFS patients significantly reduced NO production in human endothelial cells, with decreased NO production linked to higher inhibitory eNOS phosphorylation at Thr495 at the basal state. This finding provides direct experimental evidence that circulating factors in ME/CFS impair eNOS‐dependent NO production and is consistent with the eNOS uncoupling mechanism proposed here, though it does not confirm it. The neuronal isoform of nitric oxide synthase (nNOS) may additionally contribute to certain manifestations of this and related phenotypes. nNOS is expressed in peripheral neurons and the central nervous system and has been implicated in some neurological sequelae of viral illness, including anosmia, ageusia, and cognitive symptoms grouped under neuro‐COVID. Whether nNOS dysfunction in this context shares mechanistic features with the vascular eNOS uncoupling described here, particularly given that both isoforms are BH4‐dependent, represents a speculative but potentially important extension of the proposed framework. In the absence of direct evidence specific to this phenotype, this extension is offered as a hypothesis‐derived possibility requiring independent investigation and is not incorporated into the core model presented here. These phenotypes may coexist, overlap, or transition between mechanisms over time, and future biomarker studies should be designed to stratify patients by phenotype before drawing mechanistic conclusions.

### 9.6 Subclinical Endothelial Oxidative Stress: A Proposed Spectrum and Its Long‐Term Implications

The vascular‐PEM phenotype described in this paper represents the severe and clinically overt end of what may be a broader spectrum of post‐COVID endothelial perturbation. A proportion of individuals with SARS‐CoV‐2 exposure may carry subclinical BH4/BH2 imbalance and low‐grade eNOS uncoupling insufficient to produce overt PEM but sufficient to generate background endothelial oxidative stress. Within the proposed model, even subclinical eNOS uncoupling produces a sustained shift in the ratio of protective NO to oxidative superoxide and peroxynitrite at the vascular endothelium. Prolonged exposure to this altered redox environment even at low intensity may over time contribute to endothelial inflammation, impaired microvascular homeostasis, and accelerated progression of conditions whose pathophysiology depends on cumulative endothelial oxidative load. Epidemiological signals consistent with elevated post‐COVID cardiovascular, thrombotic, and autoimmune risk have been reported across multiple cohorts, though causation has not been established and confounding factors are substantial. The eNOS uncoupling framework proposed here provides one candidate biochemical mechanism through which these population‐level signals might, if confirmed, be mechanistically explained. A further implication is diagnostic. If the BH4/BH2 ratio and nitrotyrosine, the primary biomarkers proposed in Section 7, prove sensitive and specific for eNOS uncoupling in the overt phenotype, they may additionally offer a pathway toward identifying subclinical endothelial dysfunction in a broader postinfection population, before the development of clinically manifest disease. This screening application is speculative and would require large‐scale validation studies; it is offered here as a hypothesis‐derived possibility, not a clinical proposal. These broader population implications are presented as an extension of the proposed model and require independent epidemiological and biochemical investigation before any conclusions can be drawn.

### 9.7 Mechanistic Convergence With Established Vascular Biology and Therapeutic Implications

The mechanistic framework proposed in this paper is supported by independent lines of established vascular biology research that, taken together, increase the biological plausibility of the central hypothesis. The molecular cascade through which laminar shear stress activates eNOS at the apical endothelial surface has been characterized by Yang and Rizzo [52], demonstrating that β1‐containing integrins and caveolae form a mechanosensory complex transducing shear into Akt‐mediated eNOS phosphorylation. This cascade situates the glycocalyx not as an isolated structure but as the upstream component of an integrated signaling apparatus consistent with our prediction that glycocalyx restoration through sulodexide should yield clinical improvement only when paired with downstream eNOS recoupling support. The reverse phenomenon—the detrimental consequences of low or oscillatory shear—has been experimentally documented by Zhang et al. [40] and Chao et al. [11], who showed that low‐shear stress directly induces eNOS uncoupling through autophagy‐mediated phosphorylation and AT1R/ROS signaling, respectively. These findings provide direct experimental support for our prediction that complete rest may paradoxically worsen endothelial dysfunction in the vascular‐PEM phenotype by withdrawing the protective laminar shear stimulus required for KLF2‐mediated GCH1 transcription and BH4 synthesis. The concept of pharmacological substitution for absent physiological shear has been formalized by Kim and Arany [29] in the context of acute COVID‐19 and immobilized patients. Their proposal that statins, resveratrol, sulforaphane, and dipyridamole act as “shear stress mimetics” through KLF2 induction is mechanistically congruent with the Layer 4 framework presented here. The observation that low‐energy ESWT activates the identical mechanosensory complex and induces the same downstream signaling (Akt/eNOS phosphorylation, KLF2 expression) as physiological laminar shear [30] suggests that mechanical, non‐pharmacological shear stress mimetics may offer an additional therapeutic avenue for severely affected patients (Phenotype D) in whom even constant‐pace activity is not tolerated. The proposed eNOS recoupling mechanism through statins is supported by both biochemical and clinical evidence. Margaritis et al. [25] reviewed the pleiotropic effects of statins, demonstrating that beyond lipid‐lowering, they enhance eNOS expression, enzymatic activity, and coupling through mevalonate‐pathway‐dependent mechanisms while simultaneously suppressing NADPH oxidase activation through Rac1 inhibition. Sabri et al. [26] provided direct in vivo evidence that statin therapy can recouple dysfunctional eNOS in a model of acute neurovascular injury, and importantly demonstrated efficacy when administered after the inciting event relevant to chronic Long COVID where the priming insult occurred months or years before treatment initiation. Finally, the parallel between the proposed model and established vascular aging biology is notable. Sun et al. [53] demonstrated that aged mesenteric arteries exhibit reduced NO release in response to shear stress despite preserved eNOS expression, with elevated superoxide production and impaired antioxidant capacity a biochemical signature closely resembling the vascular‐PEM phenotype described here. The recovery of shear‐induced NO release with chronic moderate exercise in aged vessels provides experimental precedent for the proposed therapeutic value of sustained low‐intensity laminar shear in restoring eNOS function. The convergence of these independent lines of evidence—mechanotransduction biology, low‐shear pathology, pharmacological mimetics, statin‐mediated recoupling, and vascular aging parallels—does not validate the present hypothesis, but it situates the proposal within a broader, established framework of vascular biology rather than as an isolated speculation.

### 9.8 Alternative and Complementary Microvascular Pathways: Contextualizing the eNOS‐Centered Framework

The proposed framework prioritizes eNOS uncoupling as a candidate central node; however, the model does not propose exclusivity of eNOS‐derived NO signaling, but rather hierarchical integration within a broader endothelial dysfunction network. Microvascular regulation in resistance arteries and capillaries involves multiple endothelial signaling systems that may contribute independently of, or in parallel with, NO‐dependent pathways. Endothelium‐derived hyperpolarization (EDH), mediated through small conductance calcium‐activated potassium (SKCa) and intermediate conductance calcium‐activated potassium (IKCa) channel activation and propagated via gap junction‐dependent electrical conduction, constitutes a quantitatively important vasodilatory mechanism in vessels below 200 μm diameter—the primary anatomical territory of the proposed dysfunction. Crucially, the superoxide generated by uncoupled eNOS may directly impair both calcium‐activated potassium channels and connexin‐dependent intercellular signaling, thereby simultaneously disrupting the EDH pathway and creating a compound vasodilatory deficit beyond NO deficiency alone [54, 55]. Endothelial mechanosensory transduction of shear stress operates through transient receptor potential vanilloid 4 (TRPV4)‐mediated calcium influx, which activates SKCa/IKCa channels and initiates both NO and EDH signaling cascades. Oxidative modification of TRPV4 or the upstream platelet‐endothelial cell adhesion molecule‐1 (PECAM‐1)/vascular endothelial cadherin (VE‐cadherin)/vascular endothelial growth factor receptor 2 (VEGFR2) mechanosensory complex already incorporated into the glycocalyx framework of Section 9.7 could amplify the vasodilatory deficit independently of BH4 depletion [56, 57]. Inward rectifier potassium channels (Kir2.1) additionally contribute to conducted vasodilation across microvascular segments and may similarly be susceptible to redox impairment [58]. Mitochondrial dysfunction within endothelial cells represents an additional upstream source of reactive oxygen species capable of promoting eNOS uncoupling, calcium‐signaling abnormalities, and impaired vasodilatory reserve, and may operate as a parallel perpetuating mechanism independent of the BH4 depletion cascade [59]. Soluble guanylate cyclase (sGC), the primary effector of NO‐dependent vasodilation, is susceptible to heme oxidation producing a NO‐insensitive form; this downstream redox modification could maintain vasodilatory impairment even following partial eNOS recoupling, and represents a target addressable by direct sGC activators independent of eNOS coupling status [60]. Endothelial glycocalyx disruption documented in Long COVID and incorporated into Layer 3 of the proposed management framework impairs mechanosensory function upstream of all downstream signaling pathways including eNOS, EDH, and TRPV4 activation. Substantial heterogeneity exists between microvascular beds. In the coronary microcirculation, both NO and EDH contribute substantially to vasodilation, with EDH predominance in smaller arterioles. Cerebral microvessels depend critically on NO for neurovascular coupling, with additional regulatory complexity from astrocytic signaling. Skeletal muscle microvasculature shows high shear‐dependence consistent with the exercise‐specificity of the proposed phenotype. Renal glomerular endothelium is critically NO‐dependent for filtration pressure regulation. This heterogeneity may explain the multisystem symptom burden characteristic of Long COVID and predicts that the relative contribution of each pathway may differ across affected microvascular territories, even when the upstream oxidative environment is shared. These alternative pathways are not offered as competing explanations but as parallel dysfunction nodes that may be propagated by the same nitro‐oxidative environment proposed here. Future studies should assess their relative contributions through selective pharmacological blockade protocols. In cases where these abnormalities share oxidative stress as a contributing upstream driver, interventions restoring eNOS coupling may secondarily improve EDH function, calcium channel activity, and sGC responsiveness; however, independent contributions from mitochondrial dysfunction, connexin dysregulation, or primary glycocalyx injury may require additional targeted approaches beyond eNOS recoupling alone. A practical implication for the biomarker validation framework (Section 7) is that nitrotyrosine elevation may serve as a pathway‐agnostic marker of post‐exertional nitro‐oxidative stress across all these mechanisms, while BH4/BH2 ratio decline provides specificity for the eNOS recoupling pathway. Where nitrotyrosine is elevated post‐exertion but BH4/BH2 ratio is preserved, the data would be consistent with alternative oxidative mechanisms rather than eNOS uncoupling as the primary driver in that participant. To further distinguish these pathways, the Section 7 protocol additionally proposes reduced/oxidized glutathione ratio (GSH/GSSG) and plasma 8‐isoprostane as exploratory pathway‐agnostic oxidative stress indices.

## 10 Perspective

We propose that functional eNOS uncoupling driven by BH4/BH2 imbalance may provide a mechanistic framework connecting SARS‐CoV‐2‐induced endotheliitis to the delayed, self‐amplifying, threshold‐dependent post‐exertional malaise characteristic of a specific vascular‐PEM phenotype in Long COVID. The model additionally proposes that individual predisposition encompassing genetic variants affecting redox defense or BH4 metabolism, preexisting endothelial vulnerability, or elevated baseline eNOS expression in trained individuals may determine whether the proposed vicious cycle becomes self‐perpetuating or resolves spontaneously. In individuals without significant predisposition, the iNOS‐initiated BH4 depletion may be sufficient for the endogenous redox and recycling systems to restore BH4/BH2 balance within weeks to months, explaining the spontaneous recovery observed in a proportion of Long COVID cases at approximately 3 months. In predisposed individuals, the same initiating cascade may exceed the self‐repair capacity of DHFR and antioxidant systems, establishing a self‐amplifying loop that does not resolve without external intervention targeting the proposed pathway nodes. The critical interval between acute infection and the first overt PEM crash during which the BH4 pool is silently depleted may therefore represent a mechanistic window in which pharmacological stabilization, before the uncoupling cycle becomes entrenched, could potentially alter the long‐term trajectory of the syndrome. The proposed microvascular pathology may be invisible to standard diagnostic workups, potentially generating a diagnostic gap that frequently leads to misattribution as dysautonomia or functional disorder. Addressing this proposed gap requires diagnostic tools capable of resolving microvascular dysfunction including post‐occlusive reactive hyperaemia (PORH), flow‐mediated dilation (FMD), peripheral arterial tonometry (EndoPAT/RHI), finger thermal monitoring, invasive coronary physiology for microvascular angina assessment, cardiac stress MRI, and PET coronary flow reserve and symptom‐contingent activity frameworks that account for the mechanistic distinction between laminar and oscillatory shear.

The Two‐Threshold Model proposes that the PEM threshold and the shear stress tolerance threshold are mechanistically independent variables requiring simultaneous management. This distinction may help explain why complete rest may paradoxically worsen outcomes in some patients, why identical heart rates can produce radically different proposed endothelial outcomes depending on activity pattern, and why hemodynamic quality, specifically the distinction between laminar and oscillatory shear, is proposed as an independent determinant of endothelial outcome that activity intensity alone does not capture.

The model generates specific, falsifiable predictions testable in a prospective case–control study with dynamic pre/post‐exertion biomarker measurement. If the proposed post‐exertional biochemical signature is demonstrated, this framework would provide mechanistic justification for phenotype‐specific rehabilitation protocols that go beyond symptom‐based pacing to consider the hemodynamic quality of prescribed activity.

## Funding

The authors have nothing to report.

## Ethics Statement

The authors have nothing to report.

## Conflicts of Interest

The authors declare no conflicts of interest. Y.K.K. is the founder of EnduroMetrics, a sports performance software company. This work received no funding from the company, and no company software or product was used in this study. 

## Data Availability

Data sharing is not applicable to this article as no datasets were generated or analyzed during this study.
